# Impacts of the Callipyge Mutation on Ovine Plasma Metabolites and Muscle Fibre Type

**DOI:** 10.1371/journal.pone.0099726

**Published:** 2014-06-17

**Authors:** Juan Li, Paul L. Greenwood, Noelle E. Cockett, Tracy S. Hadfield, Tony Vuocolo, Keren Byrne, Jason D. White, Ross L. Tellam, Horst Joachim Schirra

**Affiliations:** 1 CSIRO Animal, Food and Health Sciences, St Lucia, Brisbane, Australia; 2 CSIRO Animal, Food and Health Sciences, FD McMaster Laboratory, Armidale, Australia; 3 New South Wales Department of Primary Industries, Beef Industry Centre of Excellence, University of New England, Armidale, Australia; 4 Department of Animal, Dairy and Veterinary Sciences, Utah State University, Logan, Utah, United States of America; 5 The University of Melbourne, School of Veterinary Science, Parkville, Australia; 6 The University of Queensland, Centre for Advanced Imaging, Brisbane, Australia; National Research Council of Italy, Italy

## Abstract

The ovine Callipyge mutation causes postnatal muscle hypertrophy localized to the pelvic limbs and torso, as well as body leanness. The mechanism underpinning enhanced muscle mass is unclear, as is the systemic impact of the mutation. Using muscle fibre typing immunohistochemistry, we confirmed muscle specific effects and demonstrated that affected muscles had greater prevalence and hypertrophy of type 2X fast twitch glycolytic fibres and decreased representation of types 1, 2C, 2A and/or 2AX fibres. To investigate potential systemic effects of the mutation, proton NMR spectra of plasma taken from lambs at 8 and 12 weeks of age were measured. Multivariate statistical analysis of plasma metabolite profiles demonstrated effects of development and genotype but not gender. Plasma from Callipyge lambs at 12 weeks of age, but not 8 weeks, was characterized by a metabolic profile consistent with contributions from the affected hypertrophic fast twitch glycolytic muscle fibres. Microarray analysis of the perirenal adipose tissue depot did not reveal a transcriptional effect of the mutation in this tissue. We conclude that there is an indirect systemic effect of the Callipyge mutation in skeletal muscle in the form of changes of blood metabolites, which may contribute to secondary phenotypes such as body leanness.

## Introduction

The intergenic Callipyge mutation in sheep causes large changes in selected skeletal muscles. Specific skeletal muscles in sheep of the N^mat^C^pat^ genotype (N and C being the wild type and Callipyge alleles, respectively) are increased in size by as much as 35% [1]–[4]. In an unusual genetic mechanism, termed polar overdominance, only heterozygous animals (N^mat^C^pat^) that inherit the mutation from their sire express the muscling phenotype. The maternal heterozygote (C^mat^N^pat^) and homozygote (C^mat^C^pat^) animals show no phenotype due to a complex interplay between genomic imprinting and trans-acting effects of non-coding RNA located in the same genomic region as the mutation [5]–[7]. The Callipyge allele in the N^mat^C^pat^ genotype is paternally expressed while the wild type allele is maternally imprinted and not expressed.

The Callipyge mutation causes postnatal skeletal muscle hypertrophy and a shift towards more glycolytic muscle fibres in some muscles [6], [8], [9]. The phenotype is first expressed 1–3 months post-birth and occurs along a rostro-claudal gradient in the affected N^mat^C^pat^ animal with greatest impact on skeletal muscles innervated through lumbar and sacral roots *e.g. longissimus dorsi* and *semimembranosus* muscles [4], [10]–[14]. There are also decreases in the proportional sizes of several non-muscle organs, as well as increased animal leanness and improved feed conversion efficiency but live-weight is unaffected [1]–[3], [6], [8], [9], [12].

The Callipyge point mutation has been located in a 12 bp conserved motif positioned near the telomeric end of chromosome 18 between the protein encoding gene *DLK1* and the non-protein encoding gene *GTL2* (*MEG3*) [5], [15], [16]. The broader 1 Mb region surrounding the mutation contains a cluster of conserved imprinted genes [17]–[22]. The expression pattern of a core group of these imprinted genes is strikingly perturbed in affected skeletal muscles from N^mat^C^pat^ lambs but is normal in unaffected muscles or the other genotypes [8], [9], [18], [23]–[29]. These results clearly link the altered expression of genes from this imprinted locus with the muscling phenotype.

The enhanced leanness and feed conversion efficiency in N^mat^C^pat^ lambs are indicative of shifts in systemic metabolic processes. In particular, skeletal muscles affected by the mutation are likely to be more dependent on glycolytic metabolism for energy generation as a result of a shift toward fast twitch glycolytic fibres, although the details of specific fibre type changes are not yet clear [8]–[10]. The mutation may therefore cause indirect effects in other tissues through changes in blood metabolites or endocrine factors derived from the affected muscles. To investigate the former possibility we hypothesised that there are genotype-specific changes in metabolite levels in circulating blood which reflect the changed metabolism in affected muscles and/or the metabolism in other tissues.

Metabonomics as a discipline is the investigation of the dynamic metabolic responses of living systems to a stimulus or genetic modification [30], and metabolomics is the characterisation of the metabolite composition of a biological sample [31]. Both disciplines essentially focus on the analysis of low molecular weight metabolites and attempt to assess the whole metabolic profile of a biological system. For reasons of simplicity we will use the term “metabolomics” throughout this publication. Nuclear magnetic resonance (NMR) spectroscopy is one of the major techniques used in metabolomics. The advantage of using NMR over other techniques such as mass spectrometry is that it is highly reproducible, inherently quantitative, non-destructive and requires only minimal sample preparation [32]. NMR spectra contain signals from a large number of metabolites that are observed simultaneously, and provide untargeted coverage of the major metabolites [32], [33]. NMR-based metabolomics has been particularly successful in describing subtle changes in the metabolic signatures in urine and plasma caused by mutations or a variety of physiological factors [34]–[37], including elucidating sub-pathological changes in ruminants [38]. Thus, we employed NMR-based metabolomics in the present study to assess system-wide differences in plasma metabolites from N^mat^C^pat^ and N^mat^N^pat^ sheep (which represent the two different phenotypes) at two postnatal developmental time points, 8 and 12 weeks, when the muscling phenotype and changes in expression of genes flanking the site of the Callipyge mutation were apparent in affected muscles from the N^mat^C^pat^ sheep compared to the N^mat^N^pat^ genotype. We demonstrate that postnatal lamb age affects the plasma metabolic profile and that the impact of the Callipyge mutation on plasma metabolites could only be discerned at the later age. The genotype-related metabolite changes were consistent with increased emphasis on glycolytic metabolism in affected skeletal muscles from N^mat^C^pat^ lambs although indirect additional contributions from nonmuscle tissues were also possible.

## Materials and Methods

### Ethics Statement

All animals were sampled for blood and euthanized in a humane manner in accordance with approved Utah State University Animal Care and Use Committee protocols.

### Animals and Plasma Samples

Planned matings within a flock of Dorset/Suffolk/Rambouillet cross-bred sheep were conducted to produce Callipyge (N^mat^C^pat^) and normal (N^mat^N^pat^) genotypes. All lambs were genotyped for the Callipyge SNP on chromosome 18 [15], [16]. Lambs had not been weaned but were given *ad libitum* access to a pelleted alfalfa hay and whole barley grain diet. A trace mineral salt mix was also available for *ad libitum* consumption. Blood samples from lambs at 8 and 12 weeks of age were collected into vacutainer tubes containing EDTA. The tubes were gently inverted and centrifuged to isolate the plasma, which was then stored at −80°C. The latter age is associated with full development of the muscling phenotype in Callipyge sheep although even at 8 weeks of age the phenotype is discernable [1]–[4]. Seventeen N^mat^C^pat^ lambs (8 male; 9 female) and 22 N^mat^N^pat^ lambs (12 male; 10 female) were sampled at both time points. Two blood samples (N^mat^C^pat^) at 8 weeks of age were unavailable. Lambs were weaned after 12 weeks of age.

### Muscle Histochemistry

#### Muscle sample preparation

Muscle samples (∼1 cm^2^×2 cm deep) were excised from 4 N^mat^C^pat^ and 4 N^mat^N^pat^ lamb carcasses at 72–78 days of age. These lambs were a subpopulation of the lambs whose plasma samples were analysed. Any subcutaneous fat and fascia and approximately 5 mm of muscle were removed from the superficial (dorsal) surface, leaving a block of muscle approximately 1 cm^3^ for immunohistochemistry and myofiber analyses. During sample preparation, the alignment of the sample was maintained to allow cryosections to be cut perpendicular to the direction of the muscle cells. Each muscle sample was mounted using gum tragacanth (Sigma Chemical Company, St Louis, MO: prepared 5% w/v in ddH_2_O) onto a cork block, with muscle fibres running perpendicular to the cork block. Samples were frozen by immersion in iso-pentane cooled to approximately −160°C in liquid nitrogen, before storage at −70°C.

#### Muscle fibre immunohistochemistry

Cross-sectional, 10 µm-thick, serial sections were cut from each frozen sample using a cryostat microtome (ThermoShandon AS 620 Cryostat SME, Thermotrace Ltd, Noble Park, Victoria, Australia). The sections were air dried and stored at −20°C until commencement of the staining procedures. They were then thawed, fixed in 100% acetone, and recovered in 0.01 M phosphate buffered saline, pH 7.2 (PBS) before application of blocking solution (10% non-immune serum, Zymed Laboratories, South San Francisco, CA) for 10 min. The blocking solution and subsequent reagents were applied within a well created around each tissue section using a hydrophobic slide marking pen. A volume of 50 µL of diluted monoclonal antibody against type 1 (clone WB-MHCs, Novocastra, Newcastle upon Tyne, UK; diluted 1∶100 in PBS), fast or type 2 (clone MY-32, Sigma; diluted 1∶1200 in PBS), or types 1, 2X and 2B (clone S5 8H2; diluted 1∶1000 in PBS) [39]–[41] myosin heavy chain (MHC) isoforms was applied to serial tissue sections and incubated for 1 h at 37°C in a humid chamber. Rabbit anti-laminin antibody (Sigma; diluted 1∶500 in PBS) was also included in the solution containing anti-type 1 MHC to allow cellular margins to be delineated. The antibodies were detected using a broad spectrum labeled-streptavidin-biotin amplification system and the substrate chromagen, diaminobenzidine (Zymed Laboratories, South San Francisco, CA). The stained sections were then dehydrated and cleared using graded ethanols and xylenes, and coverslips applied using a xylenes-based mounting medium.

#### Myofiber classification and morphometry

Microscopic image analysis was used to classify and measure myofibers. An Axioskop2 Plus microscope fitted with Plan-Neofluar objectives and a Zeiss Axiocam digital camera (Carl Zeiss Pty Ltd, Göttingen, Germany) was used to produce images. Images were generated using a 10× or 20× objective, depending on the size of the myofibers, and were captured using Zeiss Axiovision AC release 4.4 software, and analysed using Zeiss KSRun version 3.0 software (Carl Zeiss Pty Ltd). Myofibers were classified visually from images generated using the software program as types 1, 2C (type 1 and/or type 2A intermediate), 2A, 2AX (type 2A and/or type 2X intermediate) and 2X based on their staining characteristics for the three antibodies against muscle fibre myosin heavy chains (MHCs) [40], [42]–[44]. The imaging program was calibrated for cross-sectional area (CSA) measurements using a stage micrometer (Leica Microsystems GmbH, Wetzlar, Germany) and the cross-sectional areas of cells of each myofiber type were determined by tracing the anti-laminin-stained margins of the cells using an electronic drawing tablet. Only cells that appeared to be cut perpendicular, not oblique, to the length of the myofibers were measured for CSA.

Myofiber types previously described as 2B and 2AB [40] were classified as types 2X and 2AX on the basis that antibody S5-8H2 binds to type 2B and type 2X MHCs [41], but that limb and trunk muscles of ruminants express type 2X MHC and little or no type 2B MHC [39], [41], [45]–[47]. The most oxidative fibre type is slow twitch type 1 while the most glycolytic fibre type is fast twitch 2X. For each sample classified, the average CSA for all myofibers was calculated from the percentage and average size of the myofiber types. Effects of genotype (N^mat^N^pat^ and N^mat^C^pat^) on myofibre characteristics were tested using analysis of variance in Genstat V10 (VSN International Ltd, Hemel Hempstead, UK). Effects were considered significant at *P*<0.05. Four animals of each genotype were analysed for *semitendinosus* and *supraspinatus*, and three of these animals of each genotype were analysed for *semimembranosus.*

### NMR Spectroscopic Analysis of Plasma Samples

Plasma was thawed at room temperature and 300 µL was mixed with 100 µL of 1 M phosphate buffer (pH 7.4), 150 µL H_2_O and 50 µL D_2_O. Precipitates were removed by centrifugation (12,000×*g*) for 5 min, and 550 µL of the supernatant was transferred into 5 mm NMR tubes. ^1^H NMR spectra were recorded on a Bruker AV500 NMR spectrometer operating at a ^1^H frequency of 500.13 MHz and equipped with a 5 mm self-shielded z-gradient triple resonance probe and a B-ACS 60 sample changer (Bruker Biospin, Rheinstetten, Germany).

For each plasma sample two types of proton (^1^H) NMR spectra were acquired at 298 K. (i) 1D NOESY spectra were acquired with the *noesypr1d* pulse sequence ((RD)−90°−*t_1_*−90°−*τ_m_*−90°−acq) (Bruker Biospin pulse program library), as described previously [38], [48]. The water signal was suppressed by continuous wave irradiation during both the relaxation delay of 2.3 s and the mixing time (*τ_m_*) of 100 ms. (ii) Water-suppressed Carr–Purcell–Meibom–Gill (CPMG) spectra were acquired with the *cpmgpr1d* pulse sequence ((RD)−90°−(τ−180°−τ)_n_−acq) (Bruker Biospin pulse program library). A fixed spin-spin relaxation delay 2 nτ of 60 ms duration (τ = 500 µs) was used to eliminate the broad signals from high molecular weight analytes, and water suppression irradiation was applied during the relaxation delay of 2.3 s. For both types of spectra, 256 transients were collected into 32,768 data points using a spectral width of 20.0 ppm. All spectra were processed using TOPSPIN version 2.1 (Bruker Biospin). The free induction delays (FIDs) were multiplied by an exponential window function corresponding to 0.3 Hz line broadening factor before Fourier transformation. The acquired spectra were manually phased and baseline corrected. The spectra of plasma samples were referenced to the anomeric proton of glucose (δ = 5.233 ppm).

The assignment of peaks to specific metabolites (Table S1) was based on the online databases Human Metabolome Database and Biological Magnetic Resonance Bank [49], [50]. Assignments were confirmed by 2-dimensional (2D) ^1^H−^1^H double-quantum filtered correlation spectroscopy (DQF–COSY), ^1^H−^1^H total correlation spectroscopy (TOCSY), ^1^H−^13^C heteronuclear single quantum coherence (^13^C−HSQC) and ^1^H−^13^C heteronuclear multiple bond correlation (^13^C−HMBC) spectra measured on selected samples. The 2D NMR spectra were acquired on a Bruker Avance 900 spectrometer operating at a ^1^H frequency of 900.13 MHz, equipped with a 5 mm self-shielded z-gradient triple resonance cryogenic probe. In all 2D spectra, the ^1^H carrier frequency was positioned on the water resonance. Homonuclear 2D spectra were acquired with a spectral width of 12.0 ppm in both dimensions. A total of 512 increments with 32 transients and 2048 data points in the direct dimension were recorded. The relaxation delay was set to 1 s. TOCSY experiments used a MLEV-17 spin-lock scheme of 80 ms duration for isotropic mixing. ^13^C–HSQC and ^13^C–HMBC experiments were performed with spectral widths of 12.0 ppm and 200.0 ppm in the ^1^H and ^13^C dimensions, respectively, and the ^13^C carrier frequency was set at 100.0 ppm. A total of 512 increments with 64 transients were recorded into 2,048 data points in the direct dimension, and a relaxation delay of 1.0 s was used. GARP decoupling of the ^13^C channel was used during the acquisition time. The spectra were multiplied by a squared sine bell window function shifted by π/2 along the direct and indirect dimensions before two-dimensional Fourier transformation.

### NMR Spectra Processing and Statistical Analysis

^1^H NMR spectra were automatically data-reduced to consecutive integral regions of equal width of 0.01 ppm (“buckets”), covering the range of δ = 9.0−0.5 ppm, using AMIX (Analysis of Mixtures software package, version 3.6.6, Bruker Biospin). The chemical shift regions at δ 6.00−5.60 ppm and δ 5.20−4.68 ppm were excluded from the plasma spectra to eliminate artifacts of the urea signal and of imperfect water suppression. The regions at δ 3.63−3.60 ppm, 3.23−3.20 ppm, 3.15−3.07 ppm, 2.70−2.68 ppm, and 2.58−2.53 ppm, which contained EDTA signals, were also excluded to avoid these signals affecting the multivariate statistical analysis. For each spectrum, the resulting integral regions were normalized to the total intensity of the spectrum to correct for inter-sample differences in dilution. Subsequently, the bucketed data matrices were imported into the SIMCA−P+12.0 software package (Umetrics AB, Umeå, Sweden) for multivariate statistical analysis.

Both the 1D NOESY and CPMG ^1^H NMR spectra were subjected to multivariate statistical analysis and similar results were obtained. The 1D plasma spectra were scaled by Pareto scaling to minimize the effect of dynamic range of signal intensity of plasma metabolites on the subsequent analysis. An initial principal components analysis (PCA) was performed to determine whether there were any sample outliers and to investigate inherent differences in the samples. Three plasma samples were considerably outside of the 95% Hotelling’s T2 confidence range and were therefore excluded as outliers from subsequent analyses. Two of these samples were collected from the same Callipyge animal (N^mat^C^pat^) which was likely to be diseased (dark red plasma sample color, no glucose present, and very high lactate levels). The third plasma sample (N^mat^N^pat^, 12 weeks of age) contained a high level of an unknown metabolite. Therefore, there were 36 (14 N^mat^C^pat^+22 N^mat^N^pat^) and 37 (16 N^mat^C^pat^+21 N^mat^N^pat^) plasma samples from lambs at 8 and 12 weeks of age, respectively, in the following analyses.

To maximize the distinction between classes, supervised partial least squares – discriminant analysis (PLS–DA) (Figure S1) and orthogonal partial least squares – discriminant analysis (OPLS–DA) were employed. In addition to the measured metabolite data (*X*-matrix) which are required for unsupervised methods of multivariate statistics (*e.g.* PCA), supervised multivariate methods need additional input data about the class membership of individual samples, which are provided in the form of a *Y*–table, against which the PLS algorithm performs a regression of the *X*-matrix. In our study the group identity of Callipyge and wild type animals (wild-type = 0, Callipyge = 1) and the ages of sheep (8 and 12 weeks) were used as the two columns of the *Y*–table. In SIMCA, the number of PLS components (*A*) for the model was optimized by cross validation. *R^2^X*, *R^2^Y* and *Q^2^* were used to evaluate model quality. *R^2^X* and *R^2^Y* are the fraction of the sum of squares for the selected component representing the variance of *X* and *Y* variables, respectively, and *Q^2^* is the predictive ability parameter of the model, which is estimated by cross validation. Permutation tests were performed 200 times to validate PLS–DA models (Figure S2), and CV-ANOVA was used to validate OPLS-DA models. To better interpret the results from OPLS–DA, loadings were back-transformed by multiplying by their respective standard deviation, and weights of variables were superimposed on the loadings plot as the colour scale using MATLAB software (The Mathwork, Natwick, MA) [51]. Scores plots and loadings plots combined with variable importance in the projection (VIP) values were used to interpret the various PLS–DA models [52]. The influence on *Y* of each variable in the model is termed VIP. Buckets with VIP values >1 were considered as the most relevant for explaining *Y* and these were then selected for further analysis [52]. Where more than one bucket represented the same metabolite the “duplicate” buckets were excluded from that list so that each metabolite was represented by only one bucket. This list of metabolites observed to change significantly in the multivariate analysis was then further explored using univariate statistical analysis, following a decision-tree algorithm published by Goodpaster *et al*. [53]. First, the Shapiro–Wilk test was used to assess whether a variable was normally distributed. For normally distributed metabolites, the significance was then determined by Student’s *t* test, whereas the non-parametric Mann–Whitney U test was applied to non-normally distributed variables. The Benjamini–Hochberg method was used for multiple testing correction [54], which is appropriate for exploratory studies [55]. Metabolites with both multivariate and univariate statistical significance (VIP >1 and *P*<0.05) were considered responsible for the differentiation caused by age or genotype. All univariate statistical tests were conducted in the program package R

### Metabolic Pathway Analysis

Metabolic pathway analysis, which combines pathway enrichment analysis and topology analysis, was performed by using MetaboAnalyst 2.0 [56]. Lists of those identified metabolites which significantly changed in plasma were entered. The *Homo sapiens* and *Bos taurus* libraries were selected for metabolic pathway analysis. The *Bos taurus* library is more relevant to lamb metabolism, however the *Homo sapiens* library is better annotated. Thus, both libraries were used for pathway analyses. The algorithms used for over-representation and pathway topology analyses were the hypergeometric test and out-degree centrality, respectively. Pathways were considered significant when the *P* values calculated from the enrichment analysis were less than 0.05.

### Microarray Analysis

#### Biological samples for microarray analysis

To determine whether there were any direct or indirect effects of the Callipyge mutation in other tissues, besides skeletal muscle, we examined transcriptional data for perirenal adipose tissue. Male lambs at 12 weeks of age representing the N^pat^N^mat^ and N^mat^C^pat^ genotypes (*n* = 4 per genotype) were euthanized for sampling of perirenal adipose tissue. The tissue was dissected from each animal at a pre-determined site within 15 minutes of euthanasia, snap frozen under liquid nitrogen and stored at −80°C.

#### RNA extraction, microarray transcript profiling and analysis

Total RNA was extracted from the perirenal adipose tissue samples, treated with DNase1 (Ambion), purified using an RNeasy Mini Kit (Qiagen), subjected to on-column DNase1 treatment and then assessed for its quality [17]. The Bovine Genome Array Affymetrix GeneChip, (Affymetrix, Santa Clara, CA), containing 24,072 probe sets was employed for transcript profiling. All microarray images and quality control measurements were within recommended limits. Previous studies indicated that the bovine Affymetrix microarray could be used for analysis of ovine gene expression [8]. Image data were pre-processed using MAS5, and then background corrected followed by quantile normalization. The microarray data have been deposited in NCBI’s Gene Expression Omnibus. Differential expression for each probe set was tested on log_2_ expression intensities using the statistical package for microarray analysis in the CLCBIO commercial package (http://www.clcbio.com). *P*-values were corrected for false discovery rates (FDR) using the Benjamini and Hochberg method.

### Data Deposition

All primary NMR spectra and categorical metadata were deposited in the MetaboLights database (http://www.ebi.ac.uk/metabolights/, [57]) with the accession number MTBLS77.

## Results and Discussion

### The Callipyge Mutation Changes Muscle Fibre Types in Specific Muscles

**Figure 1:**
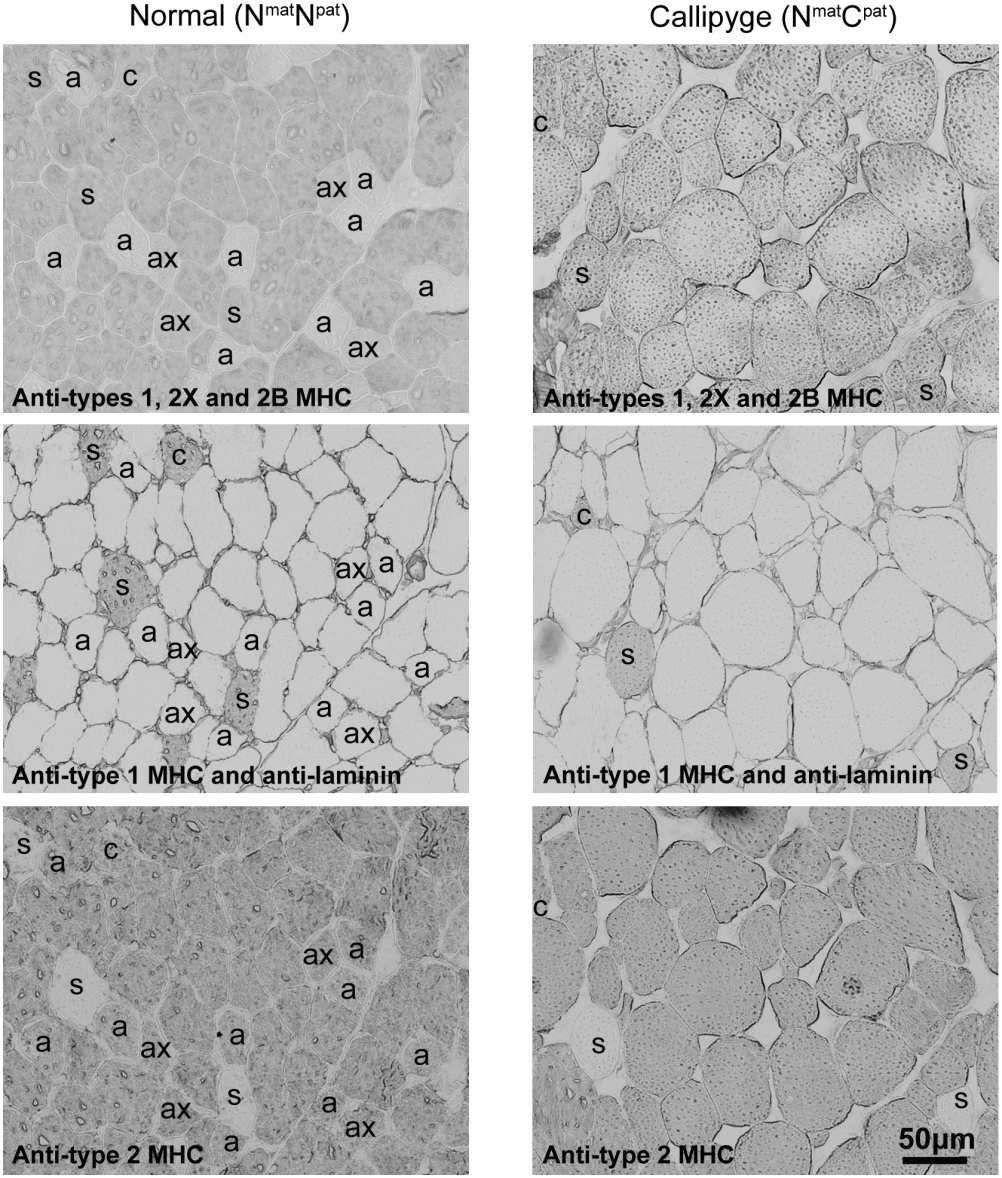
Immunohistochemical staining of *ovine* myosin heavy chains and laminin in serial sections of *semimembranosus* muscle. Representative myofibers are indicated: s, type 1 (slow oxidative); a, type 2A (fast twitch oxidative-glycolytic); c, type 2C (type 1–type 2A intermediate); ax, type 2AX (type 2A–type 2X intermediate). Non-labelled myofibers represent type 2X (fast twitch glycolytic). The top, middle and bottom panels were stained with myosin heavy chains (MHC) antibodies S5 8H2, WB-MHCs and MY32, respectively. Note the larger type 2X myofibres in the *semimembranosus* muscle of Callipyge (N^mat^C^pat^ genotype) (right column) compared with normal (N^mat^N^pat^ genotype) (left column) lambs, and the absence in the sections shown here and the overall very low prevalence of type 2A and type 2AX myofibres in Callipyge compared to normal *semimembranosus* muscle (see Table 1).

Skeletal muscle fibre type immunohistochemistry was used to examine the details of the previously reported muscle hypertrophy in Callipyge lambs at approximately 11 weeks of age (Figure 1 and Table 1) [6], [8], [9]. Each skeletal muscle typically has a mixture of fibre types that reflects the individual functional requirements of the muscle *e.g.* postural support and/or locomotion. Three functionally diverse skeletal muscles were examined to determine the effect of the N^mat^C^pat^ genotype on fibre type composition and myofibre size, namely the *semimembranosus, semitendinosus* and *supraspinatus*. Figure 1 shows representative images of the fibre type immunocytochemical staining patterns for *semimembranosus* muscle taken from N^mat^N^pat^ and N^mat^C^pat^ lambs at 11 weeks of age. This muscle is located on the back and medial side of thigh and has a primary function in locomotion. The three panels for each genotype show staining patterns in a single section, with three different monoclonal antibodies to myosin heavy chains, characteristic of specific fibre types or fibre type mixtures. These differential staining patterns of myofibre types demonstrate that *semimembranosus* is predominantly represented by type 2X fibres *i.e.* fast twitch glycolytic fibres. The predominance of type 2X fibres reflects the role of this muscle in movement, which is associated with the need for generation of force over short periods using rapidly exhaustible energy stores. Strikingly, the type 2X fibres in the N^mat^C^pat^ genotype were visibly much larger compared to the same fibre type in the N^mat^N^pat^ genotype *e.g.* see the unstained myofibres in the middle panels. There were also substantial decreases in the number of type 1, 2C, 2A and/or type 2AX fibres in the N^mat^C^pat^ genotype.

**Table 1:** Myofibre characteristics of muscle affected (*semimembranosus* and *semitendinosus*) and not affected (*supraspinatus*) by the Callipyge mutation, determined using myosin heavy chain (MHC) immunohistochemistry in normal (N^mat^N^pat^) and Callipyge (N^mat^C^pat^) lambs at 11 weeks of age.

	*Semimembranosus*				*Semitendinosus*				*Supraspinatus*			
Variable	Normal(N^mat^N^pat^)(*n* = 3)	Callipyge(N^mat^C^pat^)*(n* = 3)	s.e.d.	*P* a	Normal(N^mat^N^pat^)(*n* = 4)	Callipyge(N^mat^C^pat^)(*n* = 4)	s.e.d.	*P*	Normal(N^mat^N^pat^)(*n* = 4)	Callipyge(N^mat^C^pat^)(*n* = 4)	s.e.d.	*P*
Myofibres permm^2^ of muscleCSA^b^	499	263	85.3	0.008*	405	300	70.3	0.134	383	389	129.1	0.969
Percentmyofibres^c^												
Type 1	9.7	8.5	1.68	0.494	11.5	5.4	2.57	0.018*	33.4	36.6	8.22	0.695
Type 2C	1.9	0.8	0.51	0.028*	1.2	0.3	0.28	0.001*	5.4	4.0	2.65	0.616
Type 2A	18.2	3.6	2.09	<0.001*	19.3	6.1	2.11	<0.001*	10.1	9.0	0.90	0.219
Type 2AX	4.9	0.7	1.46	0.004*	3.3	1.7	0.78	0.044*	5.2	4.0	1.13	0.260
Type 2X	65.2	86.4	2.67	<0.001*	64.8	86.6	4.54	<0.001*	45.9	46.4	9.43	0.958
Cross-sectionalarea of myofibres(µm^2^)												
Type 1	1512	1468	135.1	0.749	1811	1282	220.2	0.016*	1931	2014	490.9	0.865
Type 2C	1041	1070	342.2	0.932	1530	2038	1006	0.614	1333	1178	380.7	0.684
Type 2A	1111	1696	147.6	<0.001*	1562	2001	545.1	0.422	1556	1640	404.9	0.836
Type 2AX	1305	1757	683.6	0.509	1789	1791	326.8	0.996	1728	2001	504.8	0.59
Type 2X	1845	3514	483.6	<0.001*	2664	2990	487.5	0.504	2160	2602	568.9	0.437
Overall	1636	3227	368.4	<0.001*	2314	2808	457.7	0.281	2018	2212	445.4	0.664

a Significant *P* values (*P*<0.05) are labeled as *.

b CSA, cross-sectional area.

c Type 1, type 1 myosin heavy chain (MHC) slow twitch oxidative fibres; Type 2A, type 2A MHC fast oxidative-glycolytic fibres; Type 2X, type 2X MHC fast twitch glycolytic fibres; Type 2C, type 1–type 2A intermediate fibres; Type 2AX, type 2A–type 2X intermediate fibres.

The size and fibre type composition in all three muscles were measured and results are presented in Table 1. Four animals of each genotype were analysed for *semitendinosus* and *supraspinatus*, and three of these animals of each genotype were analysed for *semimembranosus.* Genotype was associated with several fibre type specific changes in *semimembranosus* which was associated with a dominance of type 2X fibres in both genotypes. In Callipyge lambs the percentage of myofibres per cross-sectional area (CSA) in this muscle decreased for type 2A, 2C and 2AX fibres and markedly increased for 2X fibres compared with the N^mat^N^pat^ genotype. The CSAs for type 2A and 2X fibres were increased in Callipyge lambs indicating preferential hypertrophy of these fibres and an overall shift toward fast twitch glycolytic fibres. When all fibre types were considered there was a 1.97-fold increase in fibre cross-sectional area in Callipyge lambs. The cross-sectional area of type 1, 2C and 2AX fibres, which are more oxidative than type 2X, were unaffected in this muscle. Overall, there was a decreased number of total myofibres per CSA caused by hypertrophy of the more glycolytic fibres. The fibre type characteristics for *semitendinosus* were similar to *semimembranosus* and were likewise affected by genotype. *Semitendinosus* is also located in the rear thigh.

*Supraspinatus* is present in the upper back running between the scapula and the greater tubercle of the humerus and has roles in maintaining posture and locomotion. Fibre type analysis of this muscle showed predominant contributions from type 1 slow twitch oxidative fibres and type 2X fast twitch glycolytic fibres, which is consistent with the need for both postural and locomotive roles. Genotype had no effect on any of the fibre type characteristics of this anteriorly positioned skeletal muscle.

Overall, there was a shift in Callipyge animals towards greater prevalence and hypertrophy of fast twitch glycolytic (type 2X) fibres and loss of type 1, 2C, 2A and/or 2AX fibres in *semimembranosus* and *semitendinosus*. Collectively, these data are consistent with information demonstrating that the Callipyge mutation causes large changes in the expression of genes flanking the site of the mutation (i.e. *DLK1*, *MEG3* (*GTL2*) and also the more telomeric genes, *MEG8*, *RTL1* (*PEG11*), *RTL1AS* and *MIRG*) in *semimembranosus* and *semitendinosus* but has no effect in *supraspinatus,* and the data are also consistent with the known rostro-claudal gradient of effect of the mutation within lambs [5], [8], [9]. The affected muscles located toward the rear of the lamb are relatively large and probably represent substantial contributors to the total metabolic activity of the lamb. Hence, metabolites produced by these affected muscles may impact the composition of circulating metabolites and thereby indirectly influence other tissues not directly affected by the mutation *e.g.* adipose tissue depots.

### Plasma Metabolites are Affected by Age and Genotype, but not Gender

While a considerable number of investigations have documented the large gene expression and epigenetic changes in affected skeletal muscles associated with the Callipyge mutation [5]–[29], there has been no examination of the potential systemic metabolic impacts of the mutation. We therefore investigated metabolic changes in plasma due to the mutation using NMR metabolomics. Plasma metabolites from 17 N^mat^C^pat^ (8 male and 9 female) and 22 N^mat^N^pat^ (12 male and 10 female) lambs were investigated with NMR-based metabolomics by measuring ^1^H NMR spectra of lamb plasma samples taken at 8 and 12 weeks of age. A total of 73 plasma samples which was composed of 36 samples (14 N^mat^C^pat^+22 N^mat^N^pat^) collected at 8 weeks of age and 37 samples (16 N^mat^C^pat^+21 N^mat^N^pat^) collected at 12 weeks of age, were included in the analyses, as two blood samples (N^mat^C^pat^ at 8 weeks of age) were unavailable and three samples were excluded as extreme outliers (outside of the 95% Hotelling’s T2 confidence range).

Three potential variables were investigated that may influence plasma metabolic profiles: genotype, postnatal age and gender. To test the effects of these three factors, multivariate statistical analysis was used. Results from an initial principal components analysis (PCA) indicated that there was an effect of age independent of genotype, and an effect of genotype on plasma metabolites, but only at 12 weeks of age. No effect of gender on plasma metabolites was observed (Figure S3 and Table S2). Therefore, gender was not considered in subsequent analyses. As genotype-related effects were only visible at 12 weeks of age, we investigated the effects of age and genotype independently of each other.

### Effects of Postnatal Age on Plasma Metabolites

**Figure 2:**
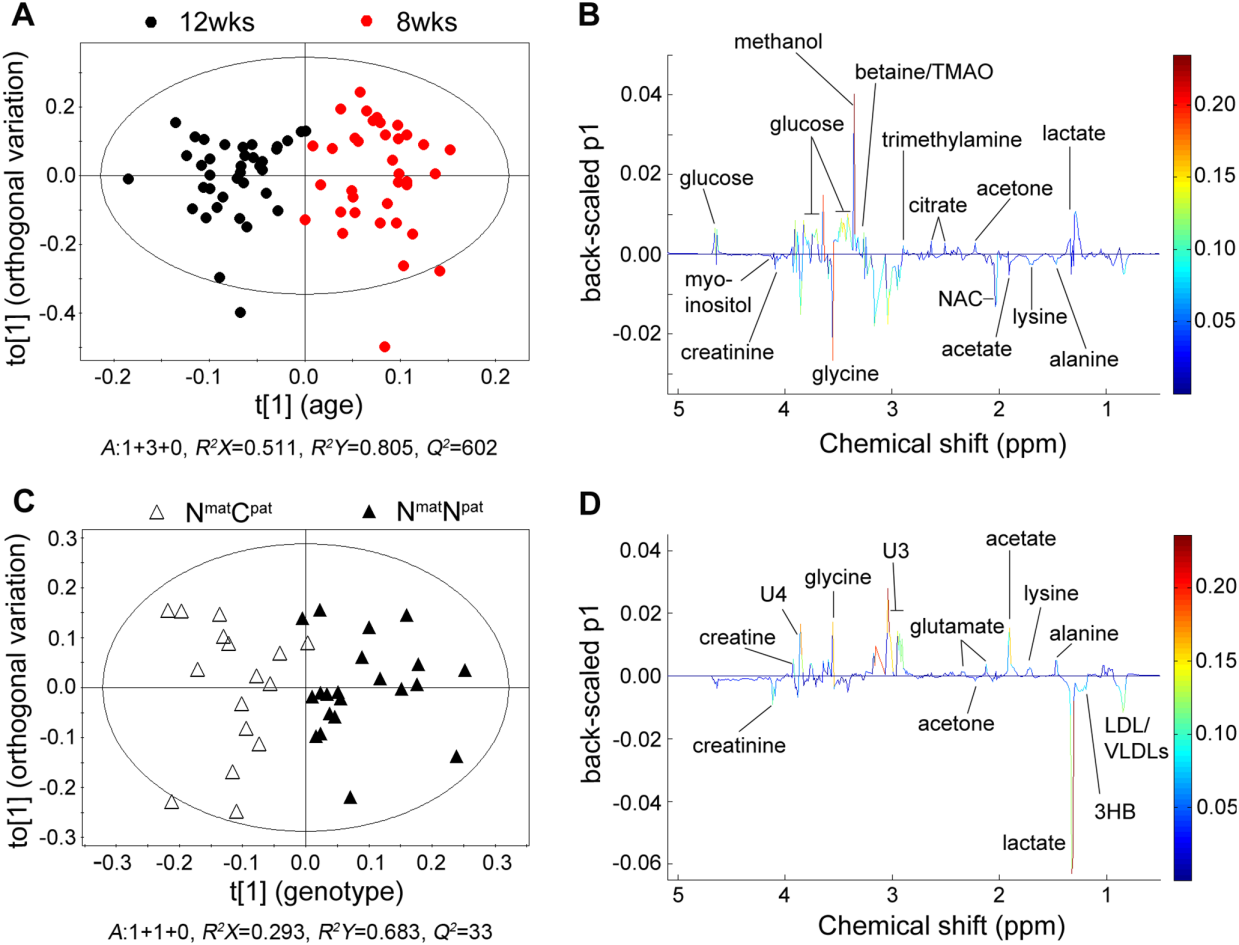
Orthogonal partial least squares – discriminant analysis (OPLS–DA) of lamb plasma metabolic profiles. Panel A: Scores plot of the comparison of plasma samples at 8 weeks (red dots) and 12 weeks (black dots) of age. Samples are separated by their age in dimension t[1], while the first orthogonal dimension to[1] contains orthogonal intra-group variation unrelated to age. Panel C: Scores plot of the comparison of plasma samples from N^mat^N^pat^ (triangles) and N^mat^C^pat^ (open triangles) lambs at 12 weeks of age. Samples are separated by genotype in dimension t[1], while the first orthogonal dimension to[1] contains intra-group variation unrelated to genotype. Panels B and D: corresponding 1D back-scaled loadings plots (0.5–5.1 ppm) for panels A and C, respectively, with the identity of several metabolites annotated. Weights of variables are shown by the colour scale. 3HB, 3-hydroxybutyrate; NAC, N-acetyl glycoproteins; TMAO, trimethylamine N-oxide; U3/U4, unknown metabolites 3/4.

To investigate the effects of postnatal age on lamb metabolism, an OPLS–DA model was computed (*n* = 73, *A*: 1+3+0, *R^2^X* = 0.511, *R^2^Y* = 0.805, *Q^2^* = 0.602, *P*(CV-ANOVA) = 2.33·10^−10^) from the 1D CPMG^ 1^H NMR spectra of plasma samples (Figure 2A and 2B). Two sets of outputs, scores plots and loadings plots, were obtained from OPLS–DA analysis of ^1^H NMR spectra. Scores plots indicate the similarity of the metabolic profiles between samples. Each data point represents one NMR spectrum (sample) and clustering of points in the plots indicates that the respective samples have similar metabolite compositions. The loadings plots illustrate the metabolites responsible for the variation within the samples observed in the corresponding scores plot [52]. The effect of age on lamb plasma metabolites was illustrated by the clustering of the samples from each age in the scores plot (Figure 2A). Plasma samples from lambs at 8 and 12 weeks of age were separated in the first component (t[1]) of the OPLS–DA, which demonstrated that the metabolic profiles of lamb plasma changed between 8 and 12 weeks of age. The results from analysis of 1D NOESY spectra presented in the Supporting Information (Figure S4) are consistent with the results obtained from 1D CPMG spectra.

To analyse intrinsic differences in the composition of the plasma metabolites between both ages, the 1D back-scaled loadings plot was investigated (Figure 2B). Levels of several metabolites were either increased or decreased with age (Figure 2B). The levels of the important metabolites (VIP >1) were subjected to univariate analysis. The Student’s *t* test or Mann–Whitney U test was performed to assess the significance of changes in metabolite concentrations over time. The data are summarized in Table 2. From 8 to 12 weeks of age, the levels of creatinine, glycine, lysine, and myo-inositol, as well as two unidentified metabolites increased with age, while the levels of acetone, betaine/trimethylamine N–oxide (TMAO), formate, glucose, methanol, and trimethylamine decreased. It is estimated that ruminants contain as many as 1×10^8^ to 1×10^10^ microorganisms (fungi, bacteria and protozoa) per mL of rumen fluid [58]. Hence, some of the identified plasma metabolites may reflect partial or exclusive contributions from the metabolic activities of rumen microorganisms.

**Table 2:** Changes in relative concentrations of key metabolites in lamb plasma during postnatal development^a^.

Metabolite	VIP^b^	8 wk	12 wk	12 wk/8 wk^c^	Significance(*P*-value)^d^	BH *P_adj_* e
acetone	1.28	0.081±0.020*^f^*	0.071±0.016^g^	0.88	1.30•10^−2*d*^	3.38•10^−2^
betaine/TMAO^h^	3.28	0.668±0.056	0.625±0.044	0.94	5.86•10^−4^	4.30•10^−3^
creatinine	1.25	0.108±0.014	0.116±0.012	1.08	9.74•10^−3^	2.68•10^−2^
formate	2.20	0.070±0.016	0.053±0.015	0.77	2.68•10^−5^	5.89•10^−4^
glucose	4.13	0.734±0.073	0.672±0.055	0.92	1.39•10^−4^	1.22•10^−3^
glycine	5.09	0.730±0.100	0.832±0.139	1.14	9.50•10^−4*d*^	5.23•10^−3^
lysine	1.44	0.135±0.013	0.145±0.012	1.07	2.27•10^−3^	7.12•10^−3^
methanol	6.37	0.315±0.274	0.153±0.023	0.49	1.38•10^−3*d*^	6.07•10^−3^
myo-inositol	1.62	0.095±0.013	0.107±0.016	1.12	6.19•10^−4*d*^	3.89•10^−3^
trimethylamine	1.70	0.110±0.015	0.097±0.019	0.88	2.12•10^−3^	7.16•10^−3^
U1^i^	1.07	0.071±0.008	0.077±0.008	1.08	1.27•10^−3*d*^	6.21•10^−3^
U2^i^	1.12	0.080±0.007	0.086±0.008	1.07	2.00•10^−3^	7.35•10^−3^

a Data were obtained by using relative intensities of plasma metabolites in 73 samples, comprising 36 (14 N^mat^C^pat^+22 N^mat^N^pat^) and 37 (16 N^mat^C^pat^+21 N^mat^N^pat^) plasma samples collected at 8 and 12 weeks of age, respectively. Concentrations of metabolites identified in Table S1, but not listed in this table, did not change significantly.

b VIP, Variable Importance in the Projection. Variables with VIP >1 were considered significant [52].

c Fold changes of metabolites in plasma at 12 weeks compared with 8 weeks of age.

d Labeled *P* values were obtained from Mann–Whitney U test and other *P* values were determined by Student’s *t* test.

e BH *P_adj_*, significance levels of metabolites after Benjamini-Hochberg multiple testing correction.

f,g Mean ± one standard deviation, *n* = 36 and 37, respectively.

h TMAO, trimethylamine N-oxide.

i Unidentified metabolites.

**Figure 3:**
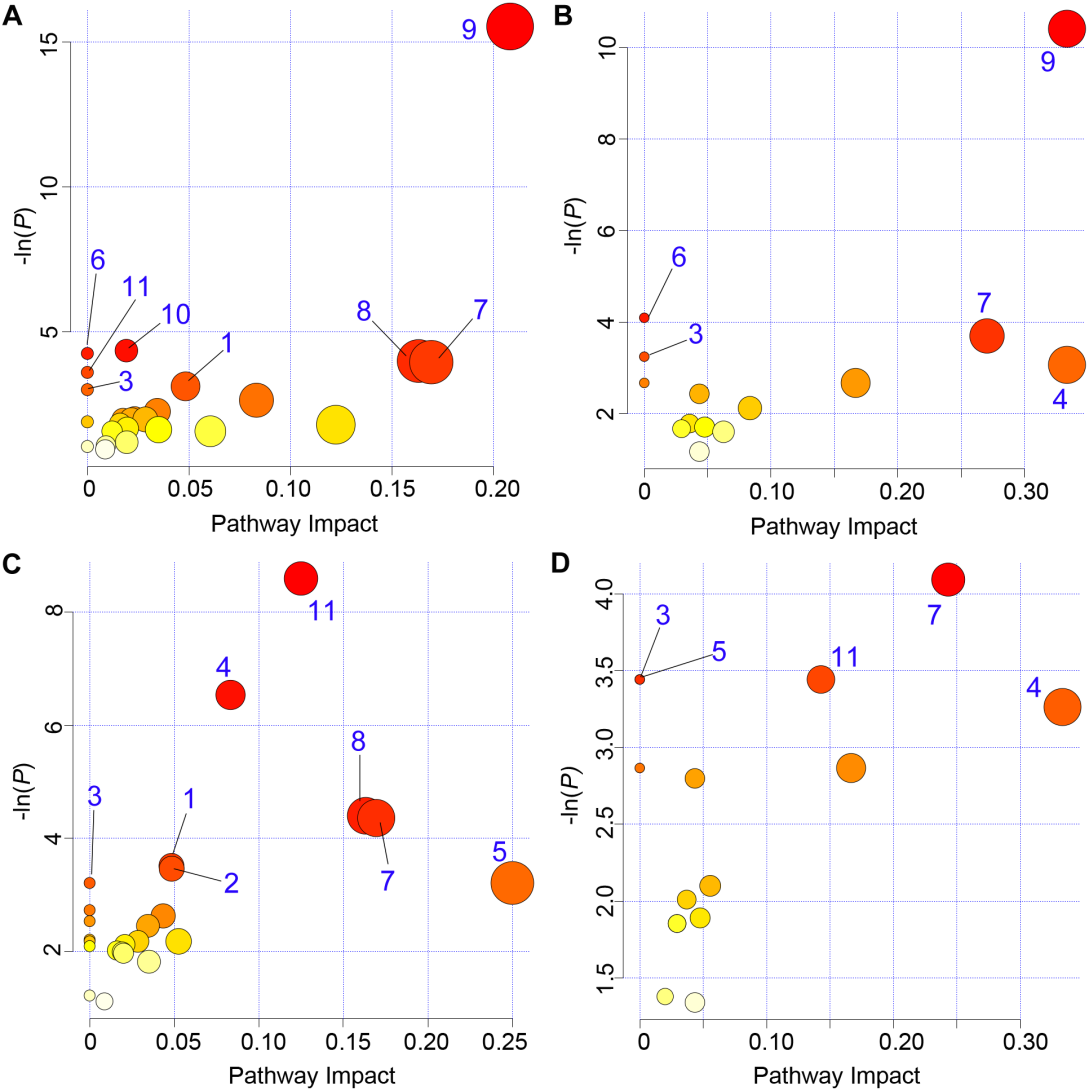
Metabolic pathway analysis. The metabolic pathways are represented as circles according to their scores from enrichment (vertical axis) and topology analyses (pathway impact, horizontal axis) using MetaboAnalyst 2.0 [56]. Darker circle colors indicate more significant changes of metabolites in the corresponding pathway. The size of the circle corresponds to the pathway impact score and is correlated with the centrality of the involved metabolites. The metabolic pathways involved in age differences are shown in panels A and B, and the pathways perturbed due to the Callipyge mutation are summarized in panels C and D. Panels A and C were generated using the *Homo sapiens* library, while the *Bos taurus* library was selected for production of panels B and D, respectively. Pathways were annotated by numbering when the *P* values calculated from the enrichment analysis were <0.05. The annotated pathways include: 1, Aminoacyl-tRNA biosynthesis; 2, Arginine and proline metabolism; 3, Biotin metabolism; 4, Cyanoamino acid metabolism; 5, D-Glutamine and D-glutamate metabolism; 6, Galactose metabolism; 7, Glycine, serine and threonine metabolism; 8, Lysine degradation; 9, Methane metabolism; 10, Nitrogen metabolism; 11, Synthesis and degradation of ketone bodies. The color of each metabolic pathway is related to the *P* value obtained from enrichment analysis and its size represents the fold enrichment score *i.e.* −ln(*P*).

Metabolic pathway analysis using MetaboAnalyst 2.0 was used to better understand the metabolic changes in lambs between 8 and 12 weeks of age. The approach employed recognises that most metabolic processes occur within cells and that plasma metabolites represent an indirect measure of these processes occurring in multiple tissues. Using the metabolites that changed between 8 and 12 weeks of age several enriched pathways (*P*<0.05) were detected (Figure 3). Using the more extensive *Homo sapiens* library (Figure 3A), the identified metabolic pathways included: methane metabolism; nitrogen metabolism; galactose metabolism; lysine degradation; glycine, serine and threonine metabolism; synthesis and degradation of ketone bodies; aminoacyl-tRNA biosynthesis; and biotin metabolism. Using the more relevant but less extensive *Bos taurus* library (Figure 3B), the enriched metabolic pathways included: methane metabolism; galactose metabolism; glycine, serine and threonine metabolism; biotin metabolism; and cyanoamino acid metabolism.

**Figure 4:**
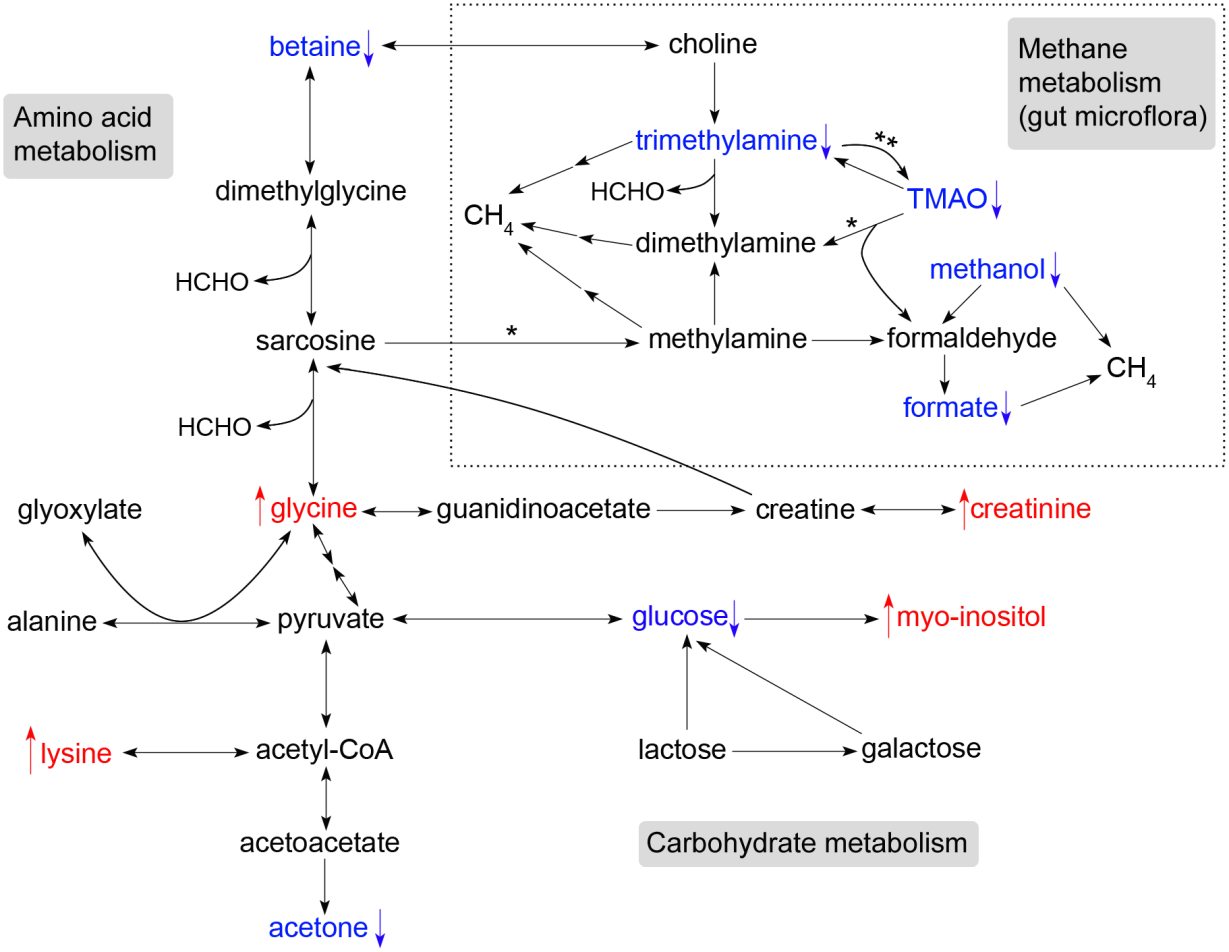
Metabolic pathways involved in postnatal lamb development. Metabolites indentified in Table 2 are summarized according to their occurrence in metabolic pathways as annotated by KEGG metabolic pathways. ↓, metabolites decreasing with age (blue); ↑, metabolites increasing with age (red). TMAO, trimethylamine N-oxide; HCHO, formaldehyde; CH_4_, methane. The reactions contained in the boxed section are likely to be derived from rumen microbial metabolism, except for reactions denoted by **, which occur in mammalian tissues [63], [84], and reactions denoted by *, which can occur both in mammalian tissues or the gut microbiome [85]. This representation of the data recognises that plasma metabolites report the combined metabolic activities of all major tissues as well as the rumen microbiome.

Figure 4 summaries the metabolic pathways that were altered in the transition from 8 to 12 weeks of age. The changes may reflect contributions from tissue metabolism as well as bacterial metabolism occurring in the rumen. Lambs were weaned after 12 weeks of age and thus there was likely to be a progressive pre-weaning diet transition including decreased intake of milk and increased solid feed intake during this period from 8 to 12 weeks of age. Consequently, the identified effects of age may be due to developmental changes in the most metabolically active tissues as well as dietary change and its associated influences on the metabolic activity of the rumen microbiota. The development of the rumen occurs in three phases: a non-ruminant phase from birth to 3 weeks; a transitional phase from 3–8 weeks; and an ‘adult’ stage beyond 8 weeks of age [59]. During the first phase the principal energy source is from metabolism of lactose and triglcerides present in ingested milk. Lambs begin ingestion of roughage during the transitional phase. Ruminants, once weaned, obtain their primary energy source in the form of volatile fatty acids generated by bacterial fermentation of ingested forage in the rumen. These fatty acids principally include acetic acid, propionic acid and butyric acid. At 8 weeks of age lambs were still transitioning into a fully functional rumen. Hence, at this age lambs were likely ingesting more milk and therefore were more reliant on lactose metabolism for energy than at 12 weeks of age. Thus, in the current study the higher level of plasma glucose at 8 weeks of age may reflect the greater reliance of these lambs on catabolism of lactose to generate energy. Consistent with our results, plasma glucose has been shown to decline with age and is further reduced by early weaning in ruminants [60].

The decrease in levels of methanol, trimethylamine, TMAO and formate in lamb plasma from 8 to 12 weeks of age may indicate changes in fermentative metabolic processes involved in establishment of the rumen microbiome during this time period. Methanol is produced in the rumen of sheep via bacterial hydrolysis of methyl esters derived from ingested pectins, the abundant polysaccharides in plants [61]. Trimethylamine can be rapidly produced by (rumen) bacterial fermentation of choline (a component of plant cell membranes) [62]–[64] or of betaine [65] and further metabolized to TMAO [66]. Methanol, trimethylamine and TMAO can also be further metabolized to formaldehyde and formate by gut microbiota [67]. Therefore, the presence of trimethylamine, TMAO, methanol and formate in plasma are likely to be primarily the result of microbial fermentation in the rumen. Plasma trimethylamine and TMAO concentrations are also associated with microbial fermentation in the gastrointestinal tracts of humans and mice [68]. Methanol, trimethylamine and formate can be further converted by bacteria to methane [69], [70]. Thus, the decreased levels of these metabolites at 12 weeks could indicate an increase in methane production by gut microflora during that period, which is likely to be associated with the establishment of the methanogenic microbial communities within the microbiota between 8 and 12 weeks of age. An additional influence of the dietary changes on the composition of the gut microbiota is also possible [71].

The reasons for the coordinated increases of plasma glycine and lysine at 12 weeks of age are unclear as these amino acids participate in several metabolic pathways. This result could suggest relatively enhanced *de novo* protein synthesis or increased deamination of amino acids by rumen microbiota within the more rapidly growing tissues at 8 weeks of age. Lysine is an essential amino acid in sheep that must be derived from either dietary protein breakdown or via the metabolic activity of rumen microorganisms [72].

Acetone levels declined with age, but two other ketone bodies, acetoacetate and 3-hydroxybutyrate, were unchanged. Thus, it is unlikely that the 8 to 12 week age transition was associated with general perturbation of the synthesis and degradation of ketone bodies.

### Effects of the Callipyge Mutation on Plasma Metabolites

As postnatal age affected lamb metabolic profiles, the analysis of effects of genotype was conducted separately at each of the two ages. PLS–DA and OPLS–DA models were calculated for the plasma samples taken from lambs at 8 and 12 weeks of age. Both PLS–DA and OPLS–DA model for samples of 8 week old lamb indicated no impact of genotype (Table S2). For the samples taken at 12 weeks of age PLS–DA analysis (Figure S1) and the OPLS–DA model (*n* = 37, *A*: 1+1+0, *R^2^X* = 0.293, *R^2^Y* = 0.683, *Q^2^* = 0.33, *P*(CV-ANOVA) = 0.01) showed genotypic differences. Correspondingly, plasma samples from the N^mat^C^pat^ and N^mat^N^pat^ lambs were separated in the scores plot (Figure 2C) in the first component (t[1]), indicating an effect of genotype. Inspection of the back-scaled loadings plot (Figure 2D) and univariate statistical analysis identified plasma metabolites characteristic of the two genotypes at 12 weeks of age (Table 3). N^mat^C^pat^ lamb plasma contained higher levels of 3-hydroxybutyrate, acetone and creatinine, and lower levels of acetate, alanine, creatine, glutamate, glycine and lysine compared with N^mat^N^pat^ lambs. There were also two unidentified metabolites whose levels were decreased in plasma of N^mat^C^pat^ lambs.

**Table 3:** Comparison of relative concentrations of metabolites in plasma from Callipyge (N^mat^C^pat^) and normal (N^mat^N^pat^) lambs at 12 weeks of age^a^.

Metabolite	VIP^b^	(N^mat^N^pat^)	(N^mat^C^pat^)	(N^mat^C^pat^)^/^(N^mat^N^pat^)^c^	Significance(*P*-value)^d^	BH *P_adj_* e
3-hydroxybutyrate	2.35	0.361±0.035*^f^*	0.400±0.057*^g^*	1.11	2.38•10^−2^	4.75•10^2^
acetate	4.29	0.423±0.131	0.311±0.069	0.73	2.09•10^−3*d*^	2.72•10^−2^
acetone	1.36	0.066±0.007	0.079±0.021	1.19	2.71•10^−2*d*^	4.70•10^−2^
alanine	1.87	0.243±0.029	0.218±0.029	0.90	1.47•10^−2^	3.48•10^−2^
creatine	2.73	0.405±0.037	0.358±0.057	0.88	8.91•10^−3^	3.31•10^−2^
creatinine	1.08	0.112±0.010	0.121±0.012	1.08	2.62•10^−2^	4.87•10^−2^
glutamate	2.03	0.224±0.025	0.198±0.027	0.88	5.82•10^−3^	3.03•10^−2^
glycine	4.37	0.885±0.134	0.763±0.116	0.86	5.36•10^−3^	3.48•10^−2^
lysine	1.52	0.194±0.017	0.179±0.017	0.92	8.81•10^−3^	3.82•10^−2^
U3^h^	4.35	1.074±0.134	0.955±0.095	0.89	3.31•10^−3^	2.87•10^−2^
U4^h^	3.80	1.336±0.157	1.226±0.107	0.92	1.35•10^−2*d*^	3.90•10^−2^

a Data were obtained by using relative intensities of plasma metabolites in 16 N^mat^C^pat^ and 21 N^mat^N^pat^ samples which were collected at 12 weeks of age from a total of 37 animals. Concentrations of metabolites identified in Table S1, but not listed in this table, did not change significantly.

b VIP, Variable Importance in the Projection. Variables with VIP >1 were considered significant [52].

c Fold changes of levels of plasma metabolites in N^mat^C^pat^ lambs compared with the levels in N^mat^N^pat^ lambs.

d Labeled *P* values were obtained from Mann–Whitney U test and other *P* values were determined by Student’s *t* test.

e BH *P_adj_*, significance levels of metabolites after Benjamini-Hochberg multiple testing correction.

f,g Mean ± one standard deviation, *n* = 21 and 16, respectively.

h Unidentified metabolites.

**Figure 5:**
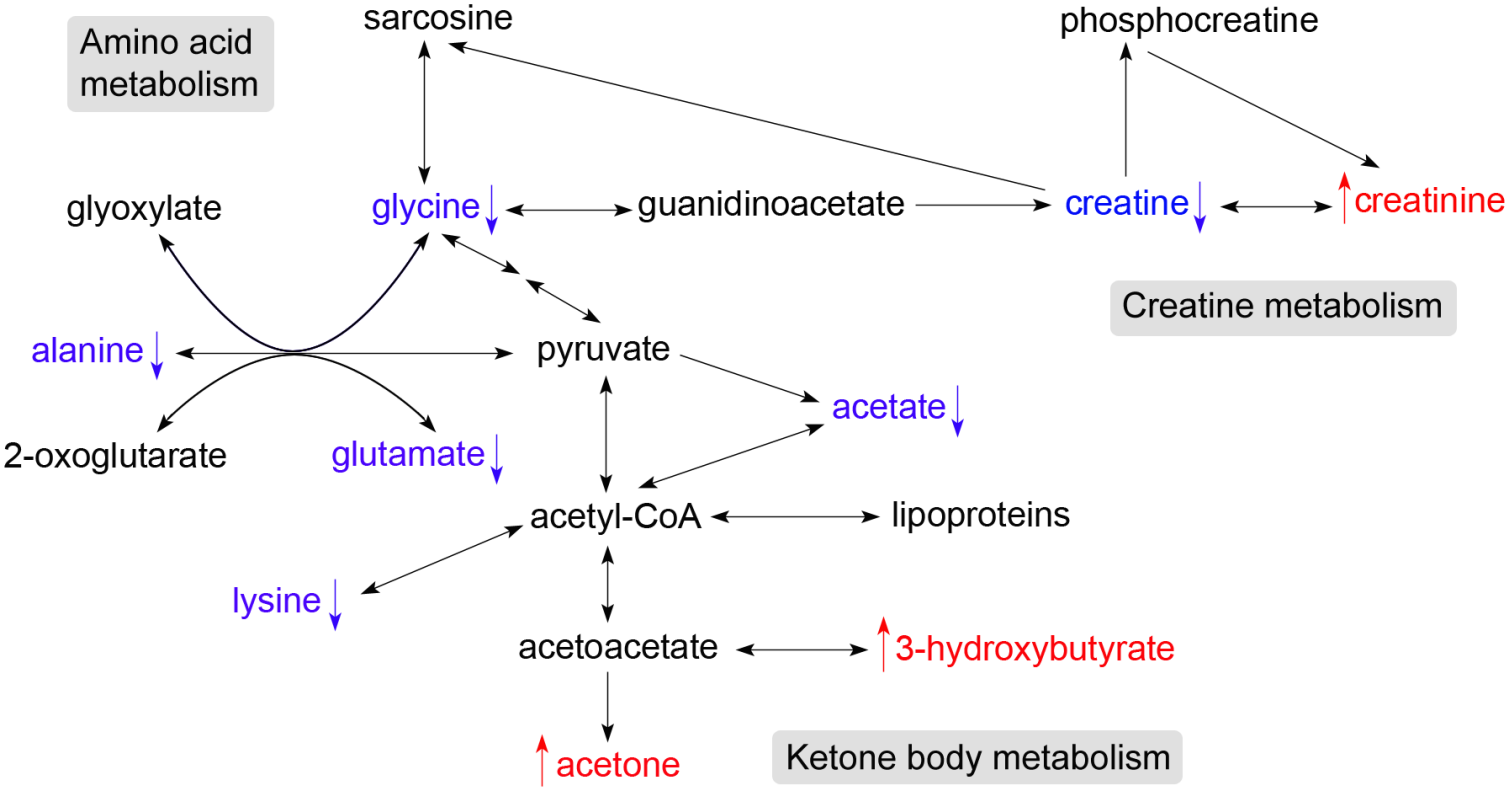
Metabolic pathways affected by the Callipyge mutation in lambs at 12 weeks of age. Metabolites indentified in Table 3 are summarized according their occurrence in metabolic pathways as annotated by KEGG metabolic pathways. ↓, metabolites decreased in plasma from N^mat^C^pat^ lambs (blue); ↑, metabolites increased in plasma from N^mat^C^pat^ lambs (red).

Metabolic pathway analysis using MetaboAnalyst 2.0 and the *Homo sapiens* library identified eight metabolic pathways altered by genotype (Figure 3C). These pathways included: synthesis and degradation of ketone bodies; cyanoamino acid metabolism; glycine, serine and threonine metabolism; biotin metabolism; D-glutamine and D-glutamate metabolism; lysine degradation; aminoacyl-tRNA biosynthesis; and arginine and proline metabolism. The latter three pathways were not detected using the *Bos taurus* library (Figure 3D). Arginine and proline metabolism pathways were altered primarily due to changes of creatine and creatinine. Thus, it is more precise to report that the Callipyge mutation was linked with change in creatine metabolism. Creatine preferentially supplies energy to skeletal muscle. The metabolic pathways impacted by the Callipyge mutation at 12 weeks of age are summarised in Figure 5.

Acetate is the most abundant volatile fatty acids in circulation and the primary metabolic substrate in ruminants. It can be taken up by most tissues and converted to acetyl-CoA for use in the TCA cycle. Hence, the reduced plasma acetate level suggests stronger energy demand in tissues from Callipyge lambs. Acetone and 3-hydroxybutyrate are ketone bodies synthesized from acetyl-CoA derived from fatty acid β-oxidation in the liver. Alternatively, they may be produced in the rumen via oxidation of butyrate, the end product of bacterial fermentation of dietary substrates [73]. Thus, the increased levels of 3-hydroxybutyrate and acetone in Callipyge lambs suggest greater utilization of dietary energy sources or enhanced mobilization and breakdown of stored fatty acids to generate energy for the hypertrophied muscles in Callipyge lambs at 12 weeks of age. In the latter case, these metabolites could be derived from the glycolytic metabolism associated with the more predominant type 2X fibres in affected muscles from Callipyge lambs. There was also a slight increase in the level of circulating lipoproteins at this age which may also reflect an enhanced mobilization of fatty acids in Callipyge lambs. These results are also consistent with the overall lean Callipyge phenotype [8]–[10].

Creatine is primarily produced from amino acids in the kidney and liver. It is transported in blood and taken up by creatine-requiring tissues, particularly skeletal muscle [74]. More than 90% of creatine is localised in muscle, and therefore changes in plasma creatine levels likely directly reflect changes in muscle metabolic requirements. Creatine plays an important role in cellular energy metabolism by interconversion to its high-energy phosphorylated analogue, phosphocreatine (PCr), catalysed by creatine kinase (CK) [74], [75]. The CK/PCr system functions as a shuttle of high-energy phosphate between ATP-generating and ATP-consuming tissues with high and fluctuating energy demand, such as in skeletal muscle, heart and brain [74]–[76]. Creatine is either taken up from ingested food and/or synthesized in kidney, pancreas and liver from arginine and glycine via a two-step reaction (Figure 5) [76]–[78]. Creatine in solution is in equilibrium with its spontaneously formed cyclic derivative creatinine, which is transferred through blood and excreted by the kidney into urine [74]. The level of plasma creatinine probably reflects the total creatine level in the body, as urinary creatinine excretion is an indicator of total creatine stores [77]. Thus, the decreased level of plasma creatine in Callipyge lambs probably signifies increased uptake by skeletal muscles affected by the mutation as a consequence of their high energy demand driven by increased reliance on fast twitch glycolytic muscle fibres. The increased level of plasma creatinine may therefore represent enhanced breakdown of creatine and phosphocreatine in muscles affected by the mutation and secretion of creatinine from the affected muscle into plasma enroute to the kidney. Increased creatinine levels associated with the Callipyge mutation may also reflect the lean body mass of these animals [79].

The decreased levels of alanine, glutamate, glycine, and lysine suggest a greater demand for protein synthesis by the hypertrophied skeletal muscles in Callipyge lambs or other changes in nitrogen metabolism affected by the mutation. Several amino acids, including alanine, glutamate and glycine, are involved in interorgan amino acid cycles that transport carbon and nitrogen between the peripheral tissues and the liver, where in the latter case they are used for urea synthesis [80]. Hence, the decreased plasma levels of these three amino acids could indicate reduced protein catabolism in these peripheral tissues in Callipyge lambs.

The impact of the Callipyge mutation on lamb plasma metabolites was only observed at 12 weeks of age. In keeping with this result the muscle hypertrophy caused by the mutation characteristically only develops over a period of approximately 2–3 months after birth [27]. Typically, the changes in expression of genes flanking the site of the mutation are first detected about one month after birth and are fully developed at three months of age. Once developed these gene expression changes persist into adulthood. It is unclear whether these phenotypic and gene expression developmental time frames are the same as those for the plasma metabolite changes. Greater time resolution in sampling is required to clarify this point. Plasma reports the combined metabolic activities of all tissues. Thus, the metabolic contribution to plasma from muscles affected by the mutation is diluted by contributions from other unaffected tissues. It is therefore possible that the impact of the Callipyge mutation induced hypertrophied muscles on plasma metabolites may vary due to differences in the ratio of hypertrophied muscle mass to total lamb body mass at 8 and 12 weeks of age.

### Transcriptional Profiling of Lamb Perirenal Adipose Tissue

The Callipyge mutation changes the expression of genes flanking the site of the mutation in specific hypertrophied muscles [8], [9], [18], [23]–[29]. Its direct effects, if any, in other tissues are unknown. The changes in plasma metabolites associated with the Callipyge mutation may be derived from metabolic changes in affected muscles or were also due to direct and/or indirect effects of the mutation in other large metabolically active tissues. The N^mat^C^pat^ genotype is linked with a postnatal rostro-claudal gradient of muscle hypertrophy as well as whole body leanness [4], [10]–[14]. The latter trait may arise as an indirect response of adipose tissue depots to the affected hypertrophied muscles or directly as a result of gene expression changes surrounding the site of the mutation in adipose tissue which drive changes in adipose tissue metabolism, deposition and released metabolites. To differentiate between these possibilities transcriptional profiling of a representative white adipose tissue depot, perirenal adipose tissue from N^mat^C^pat^ and N^mat^N^pat^ lambs at 12 weeks of age (*n* = 4/group) was performed using microarrays. This depot is metabolically active and available in sufficient quantities for analysis from the lean Callipyge sheep [81]. A total of 203 genes were differentially expressed (*P*≤0.05; fold change >1.5), although none was significant after multiple testing correction (Table S3). Thus, there were no genotype specific effects in this tissue and especially no indication of genotype specific changes for genes encoding proteins involved in adipocyte metabolism. Importantly, there were no changes in the expression of genes (*DLK1* and *MEG3*) that flank the site of the Callipyge mutation, unlike affected muscles from the N^mat^C^pat^ genotype [8]. Therefore, there were no direct effects of the mutation revealed by transcriptional analysis of this adipose tissue depot. The changes in body leanness (reduced adipose tissue deposition) and organ size associated with the mutation are likely to be indirect effects caused by the hypertrophy and changed fibre types associated with specific muscles affecting other tissues, but not the perirenal adipose tissue depot. The reason for this is not clear. The molecular communication between the affected skeletal muscles and other adipose tissue depots could be mediated by circulating metabolites arising from the hypertrophied muscles. Alternatively, communication between affected muscles and other tissues, especially adipose tissues, could be mediated by as yet undefined mutation-induced myokines [82]. Hormones such as growth hormone, insulin, thyroxine and IGF-I arising from various nonmuscle tissue sources are unlikely to play a role in this interorgan communication process as there are no changes in their levels in Callipyge lambs [83].

## Conclusions

In conclusion, several metabolic pathways are involved in plasma metabolic changes associated with the lamb developmental, dietary and rumen transitions between 8 and 12 weeks of age and with the Callipyge mutation at 12 weeks of age. During postnatal development, but not as a result of the mutation, there was also a contribution to plasma from metabolites likely derived from rumen microbiota. Muscle fibre typing in posterior but not anterior muscles indicated that the mutation caused selective hypertrophy of type 2X fibres, which are more reliant on glycolytic metabolism for generating energy. The effects of the mutation on plasma metabolites are consistent with enhanced reliance on glycolytic muscle fibres in the hypertrophic skeletal muscles affected by the mutation. The Callipyge mutation causes systemic effects on plasma metabolites which may be indirectly driving secondary Callipyge phenotypes, such as body leanness. Callipyge sheep may be particularly dependent on lipid mobilization to supply energy to their hypertrophied skeletal muscles.

## Supporting Information

Figure S1 **Partial least squares – discriminant analysis (PLS–DA) of lamb plasma metabolic profiles.** Panel A: Scores plot of the comparison of plasma samples at 8 weeks (red) and 12 weeks (black) of age. Separation according to age is noticeable in a direction between dimensions t[1] and t[2]. Panel C: Scores plot of the comparison of plasma samples from N^mat^N^pat^ (triangles) and N^mat^C^pat^ (open triangles) lambs at 12 weeks of age. Separation according to genotype is noticeable in a direction between dimensions t[1] and t[2]. Panels B and D: corresponding loadings plots for Panels A and C, respectively, with the identity of several metabolites annotated. t[1] and t[2] are the first and second PLS components, respectively. The percentage of variation explained by each component is shown in brackets.
(TIF)

Figure S2 **Validation of partial least squares – discriminant analysis (PLS–DA) by permutation analysis.** Panels A, C, E and G: Validation plots from PLS**–**DA of 1D CPMG ^1^H NMR spectra. Panels B, D, F and H: Validation plots from PLS**–**DA of 1D NOESY ^1^H NMR spectra. The age of lambs was used as the *Y* table for Panels A and B. The genotypes of lambs at 12 and 8 weeks of age were used as the *Y* table for Panels C and D, and Panels E and F, respectively. The gender of lambs was used as the *Y* table for Panel G and H. In each panel the *Y*-table of the original model (*R^2^* and *Q^2^* data point at right side of the panel) is permuted 200 times, which leads in valid models to a decrease in *R^2^* and *Q^2^* values. The vertical axes are *R^2^* and *Q^2^*, respectively, while the horizontal axes indicate how similar the permuted models are to the original model. Panels A **–** D demonstrate that development and the Callipyge genotype at 12 weeks of age affected plasma metabolites, while Panels E **–** H illustrate that the Callipyge genotype at 8 weeks of age and gender have no impact on lamb plasma metabolites. Dots and triangles are *R^2^* and *Q^2^* values obtained from permutation tests, respectively. Solid lines are *R^2^* regression lines. Dashed lines are *Q^2^* regression lines.
(TIF)

Figure S3 **Principal components analysis (PCA) of 1D CPMG ^1^H NMR spectra recorded from lamb plasma samples.** Panel A: Scores plot of the comparison of plasma samples at two developmental ages, 8 weeks (red) and 12 weeks (black), respectively. Slight separation according to age is visible in dimension t[8], while t[1] contains mainly unspecific inter-sample variation. Panel C: Scores plot of the comparison of plasma samples from N^mat^N^pat^ (triangles) and N^mat^C^pat^ (open triangles) lamb genotypes at 12 weeks of age. Genotype separation is noticeable in a direction between dimensions t[1] and t[5]. Panels B and D: Corresponding loadings plots for Panels A and C, respectively, with the identity of several metabolites annotated. TMAO, trimethylamine-N-oxide. t[1], t[5] and t[8] are the first, fifth and eighth PCA components, respectively. The percentage of variation explained by each component is shown in brackets. The separation due to development or Callipyge mutation is visible in the higher principal components (PCs). Other lower PCs were not associated with any specific biological factors in the system and were likely associated with individual variations in lambs. In order to focus on the biological questions of interest, supervised multivariate statistical analyses were performed (PLS**–**DA and OPLS**–**DA) and reported (OPLS**–**DA) in the manuscript.
(TIF)

Figure S4 **Partial least squares – discriminant analysis of 1D NOESY ^1^H NMR spectra recorded for lamb plasma samples.** Panel A: Scores plot of the comparison of plasma samples at two ages, 8 weeks (red) and 12 weeks (black), respectively. Separation according to age is visible mainly in dimension t[1], while t[2] contains mainly unspecific inter-sample variation. Panel C: Scores plot of the comparison of plasma samples from N^mat^N^pat^ (triangles) and N^mat^C^pat^ (open triangles) lambs at 12 weeks of age. Separation according to genotype is visible mainly in dimension t[1], while t[2] contains mainly unspecific inter-sample variation, next to a small amount of genotype-specific separation. Panels B and D: Corresponding loadings plots of Panels A and C, respectively, with the identity of several metabolites annotated. TMA, trimethylamine; 1, lipid = CH-CH_2_-CH = ; 2, unknown.
(TIF)

Table S1 **Resonance assignments for key metabolites.**
(DOCX)

Table S2 **The properties of multivariate statistical analysis models of plasma samples.**
(DOCX)

Table S3 **Microarray analysis of perirenal adipose tissue as a function of Callipyge genotype.**
(DOCX)
