# Supplementary figures and images for: Impacts of the Callipyge Mutation on Ovine Plasma Metabolites and Muscle Fibre Type
Source: PLoS One. 2014 Jun 17;9(6):e99726. doi: 10.1371/journal.pone.0099726 (PMC4061035; doi:10.1371/journal.pone.0099726)

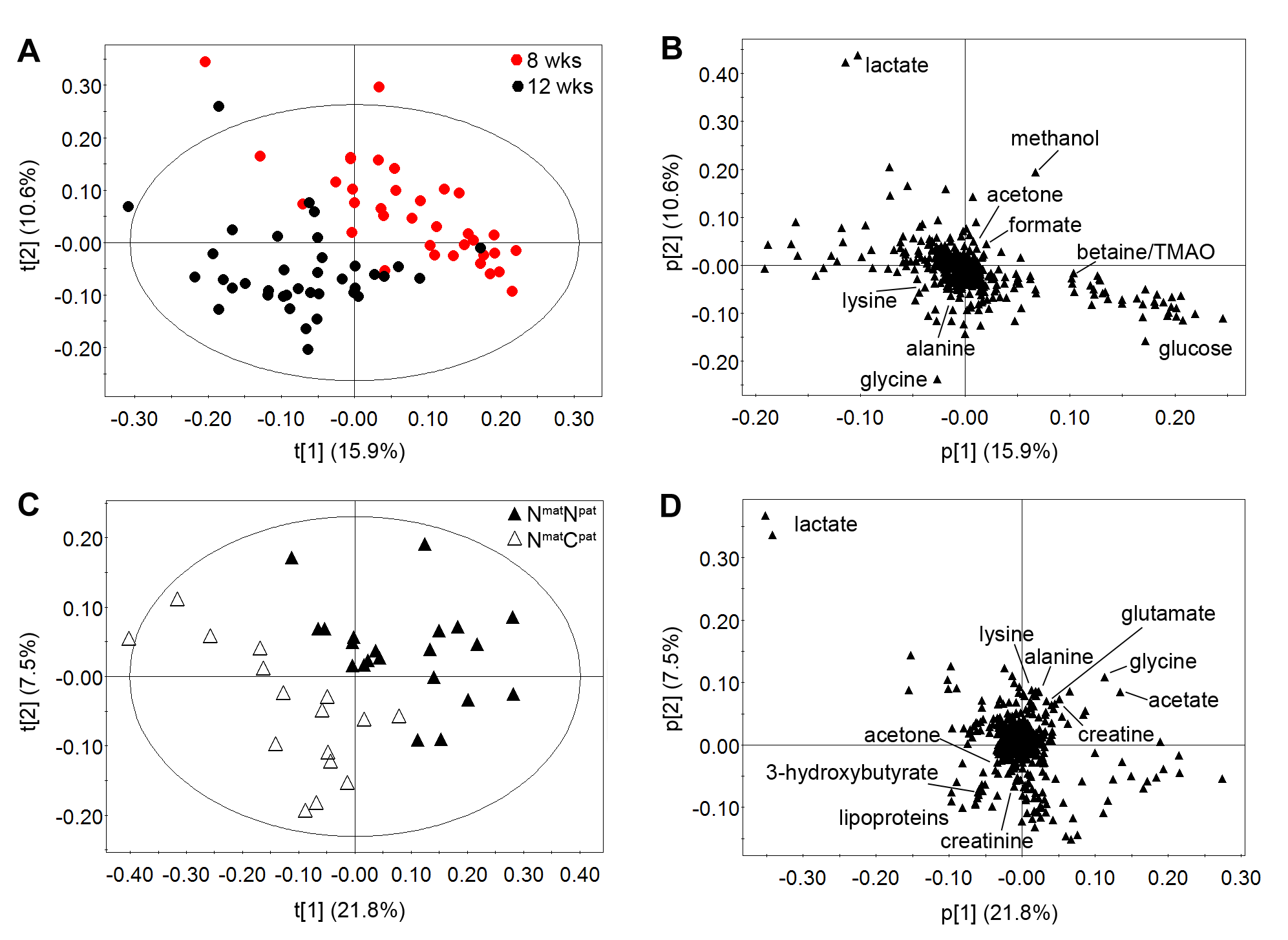

Supplement: Figure S1 — Partial least squares – discriminant analysis (PLS–DA) of lamb plasma metabolic profiles. Panel A: Scores plot of the comparison of plasma samples at 8 weeks (red) and 12 weeks (black) of age. Separation according to age is noticeable in a direction between dimensions t[1] and t[2]. Panel C: Scores plot of the comparison of plasma samples from NmatNpat (triangles) and NmatCpat (open triangles) lambs at 12 weeks of age. Separation according to genotype is noticeable in a direction between dimensions t[1] and t[2]. Panels B and D: corresponding loadings plots for Panels A and C, respectively, with the identity of several metabolites annotated. t[1] and t[2] are the first and second PLS components, respectively. The percentage of variation explained by each component is shown in brackets. (TIF) [file pone.0099726.s001.tif]

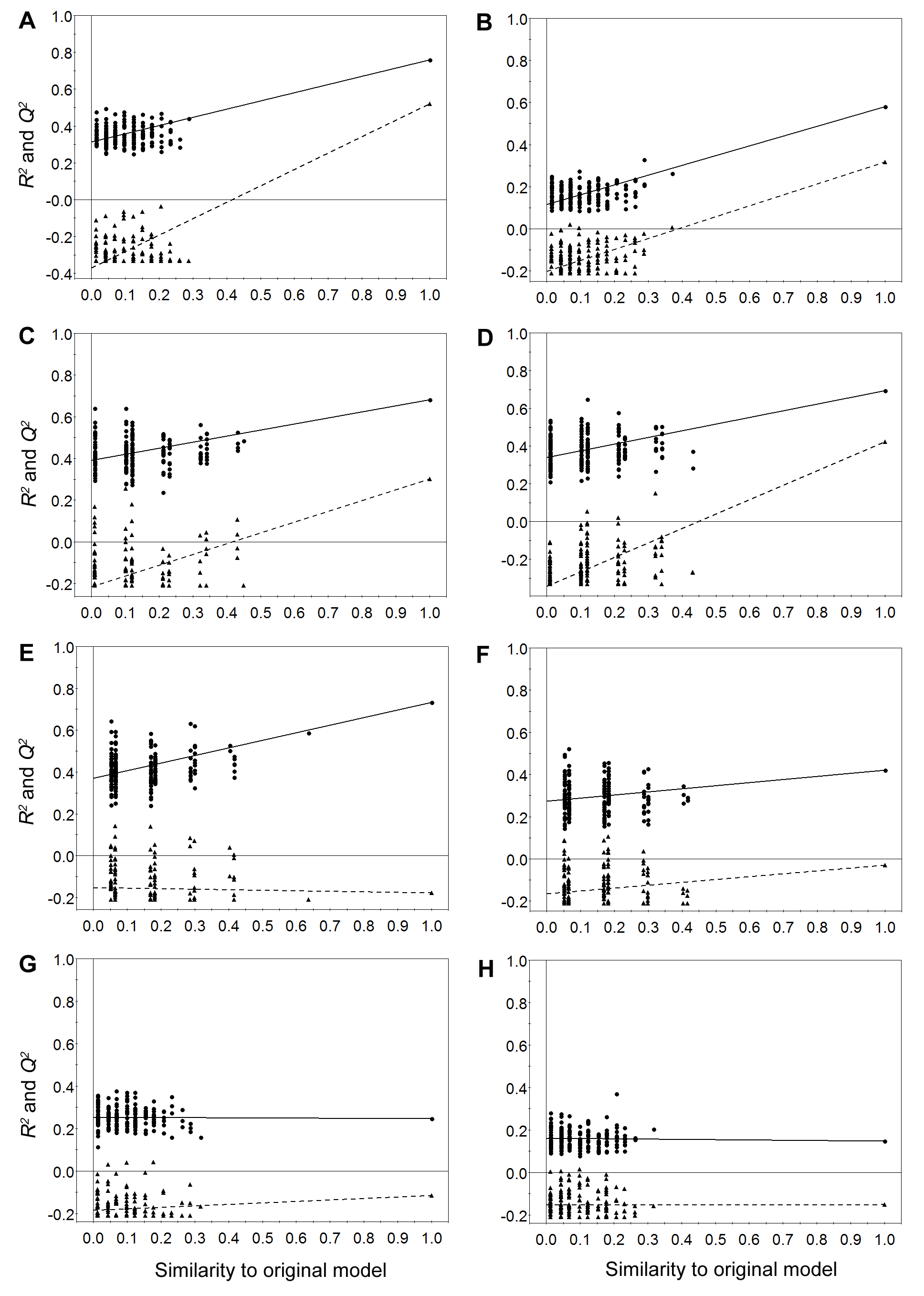

Supplement: Figure S2 — Validation of partial least squares – discriminant analysis (PLS–DA) by permutation analysis. Panels A, C, E and G: Validation plots from PLS–DA of 1D CPMG 1H NMR spectra. Panels B, D, F and H: Validation plots from PLS–DA of 1D NOESY 1H NMR spectra. The age of lambs was used as the Y table for Panels A and B. The genotypes of lambs at 12 and 8 weeks of age were used as the Y table for Panels C and D, and Panels E and F, respectively. The gender of lambs was used as the Y table for Panel G and H. In each panel the Y-table of the original model (R2 and Q2 data point at right side of the panel) is permuted 200 times, which leads in valid models to a decrease in R2 and Q2 values. The vertical axes are R2 and Q2, respectively, while the horizontal axes indicate how similar the permuted models are to the original model. Panels A – D demonstrate that development and the Callipyge genotype at 12 weeks of age affected plasma metabolites, while Panels E – H illustrate that the Callipyge genotype at 8 weeks of age and gender have no impact on lamb plasma metabolites. Dots and triangles are R2 and Q2 values obtained from permutation tests, respectively. Solid lines are R2 regression lines. Dashed lines are Q2 regression lines. (TIF) [file pone.0099726.s002.tif]

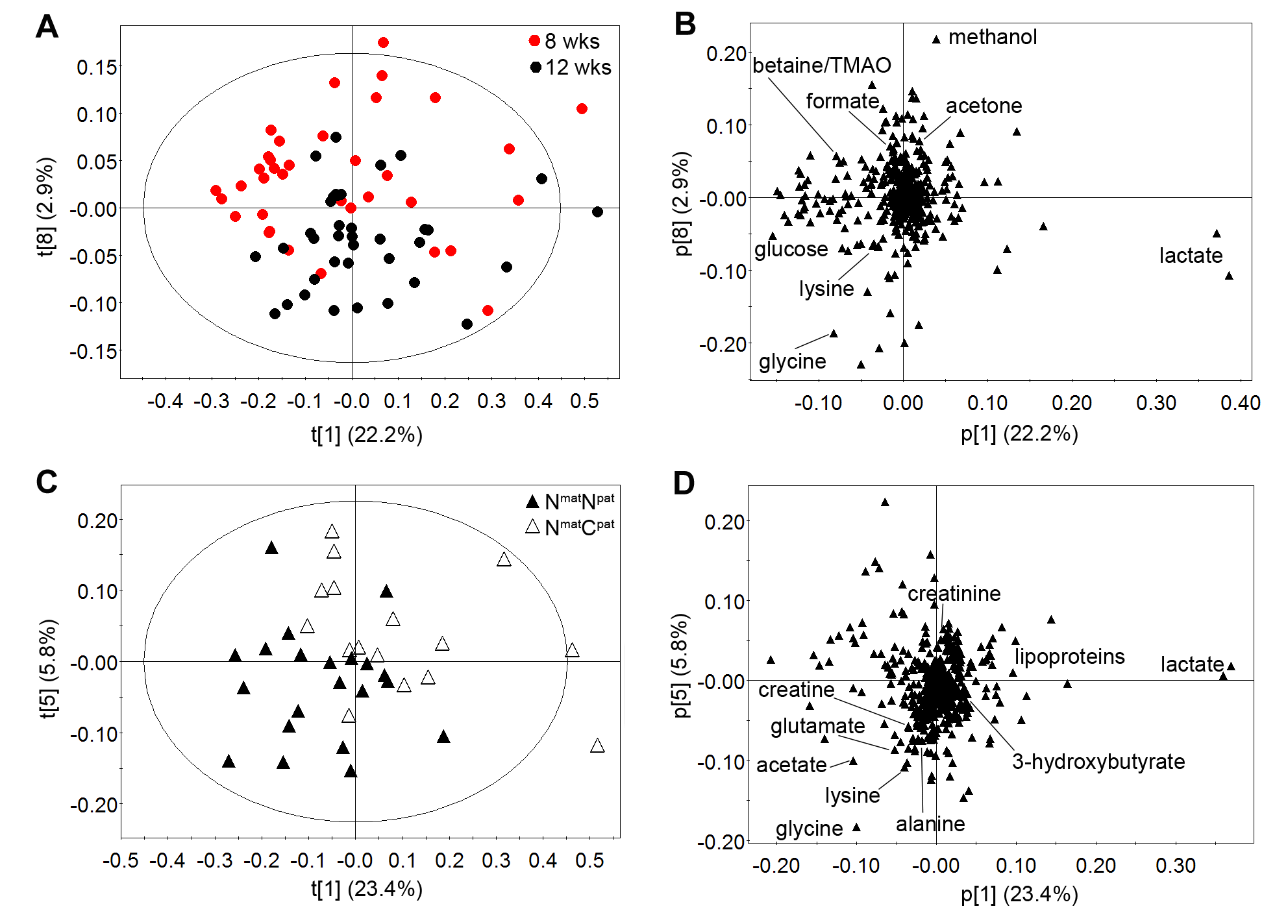

Supplement: Figure S3 — Principal components analysis (PCA) of 1D CPMG 1H NMR spectra recorded from lamb plasma samples. Panel A: Scores plot of the comparison of plasma samples at two developmental ages, 8 weeks (red) and 12 weeks (black), respectively. Slight separation according to age is visible in dimension t[8], while t[1] contains mainly unspecific inter-sample variation. Panel C: Scores plot of the comparison of plasma samples from NmatNpat (triangles) and NmatCpat (open triangles) lamb genotypes at 12 weeks of age. Genotype separation is noticeable in a direction between dimensions t[1] and t[5]. Panels B and D: Corresponding loadings plots for Panels A and C, respectively, with the identity of several metabolites annotated. TMAO, trimethylamine-N-oxide. t[1], t[5] and t[8] are the first, fifth and eighth PCA components, respectively. The percentage of variation explained by each component is shown in brackets. The separation due to development or Callipyge mutation is visible in the higher principal components (PCs). Other lower PCs were not associated with any specific biological factors in the system and were likely associated with individual variations in lambs. In order to focus on the biological questions of interest, supervised multivariate statistical analyses were performed (PLS–DA and OPLS–DA) and reported (OPLS–DA) in the manuscript. (TIF) [file pone.0099726.s003.tif]

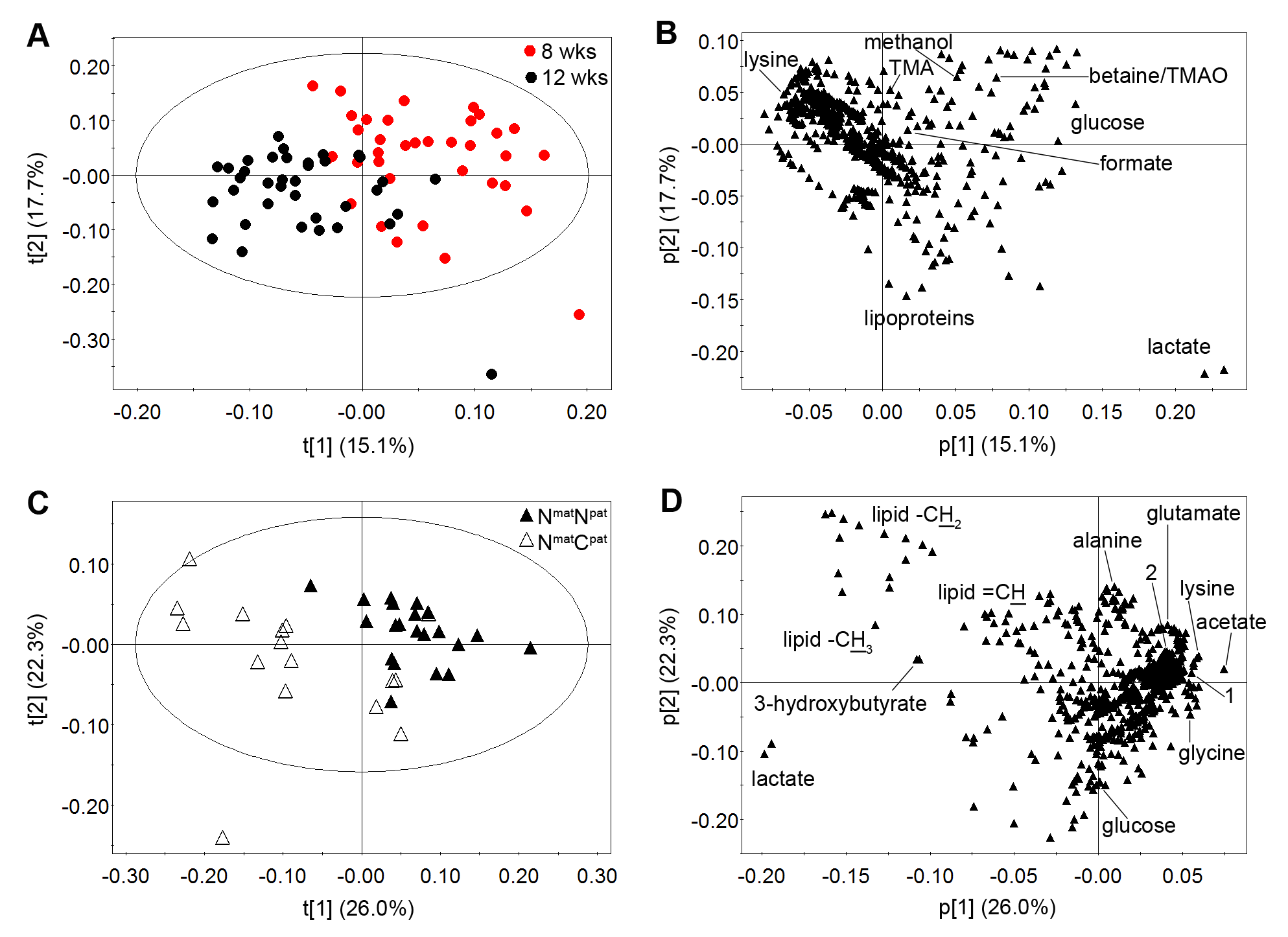

Supplement: Figure S4 — Partial least squares – discriminant analysis of 1D NOESY 1H NMR spectra recorded for lamb plasma samples. Panel A: Scores plot of the comparison of plasma samples at two ages, 8 weeks (red) and 12 weeks (black), respectively. Separation according to age is visible mainly in dimension t[1], while t[2] contains mainly unspecific inter-sample variation. Panel C: Scores plot of the comparison of plasma samples from NmatNpat (triangles) and NmatCpat (open triangles) lambs at 12 weeks of age. Separation according to genotype is visible mainly in dimension t[1], while t[2] contains mainly unspecific inter-sample variation, next to a small amount of genotype-specific separation. Panels B and D: Corresponding loadings plots of Panels A and C, respectively, with the identity of several metabolites annotated. TMA, trimethylamine; 1, lipid = CH-CH 2-CH = ; 2, unknown. (TIF) [file pone.0099726.s004.tif]
