# Supplementary material for: Impacts of the Callipyge Mutation on Ovine Plasma Metabolites and Muscle Fibre Type
Source: PLoS One. 2014 Jun 17;9(6):e99726. doi: 10.1371/journal.pone.0099726 (PMC4061035; doi:10.1371/journal.pone.0099726)
Supplement: Table S1 — Resonance assignments for key metabolites. (DOCX) [file pone.0099726.s005.docx]

**Table S1.** Resonance assignments for key metabolites.

| Metabolites^a^ | Moieties: δ ^1^H [ppm] (multiplicity)/ δ ^13^C [ppm]^b^ |
| --- | --- |
| 2-oxoglutarate | γCH_2_: 2.42(t)/33.7, βCH_2_: 3.00(t)^c^ |
| 3-hydroxybutyrate | γCH_3_: **1.19**(d)/24.47, αCH^y^H^x^: 2.31(m), 2.40(m)/49.27, βCH: 4.14(m)/68.54 |
| acetate | αCH_3_: **1.91**(s)/26.06 |
| acetone | CH_3_: **2.22**(s)^c^ |
| alanine | βCH_3_: **1.47**(d)/19.01, αCH: 3.78(q)/53.50 |
| betaine | γCH_3_: **3.25**(s)/56.08, αCH_2_: 3.90(s)/68.88 |
| Ca-EDTA^2-^ | αCH_2_: 2.56(s)/57.86, γCH_2_: 3.12(dd)/63.69 |
| citrate | CH^y^H^x^: 2.53(d), 2.65(d)/48.51 |
| creatine | γCH_3_: 3.03(s)/39.67, αCH_2_: **3.93**(s)/56.50 |
| creatinine | γCH_3_: 3.04(s)/32.96, αCH_2_: **4.05**(s)/59.22 |
| dimethylamine | N(CH_3_)_2_: 2.70(s)/35.61 |
| EDTA | αCH_2_: 3.21(s)/53.75, γCH_2_: 3.62(s)/60.00 |
| formate | CH: **8.45**(s)^c^ |
| α-glucose | 5-CH: 3.41(t)/72.49, 3-CH: 3.53(dd)/74.28, 4-CH: 3.71(t)/75.63, CH^y^H^x^: 3.77(m), 3.84(m)/63.51, 6-CH: 3.83(ddd)/74.10, 2-CH: **5.23**(d)/94.87 |
| β-glucose | 3-CH: 3.24(dd)/76.99, 5-CH: 3.40(t)/72.49, 6-CH: 3.46(ddd)/78.70, 4-CH: 3.49(t)/78.60, CH^y^H^x^: 3.73(m), 3.90(dd)/63.53, 2-CH: 4.65(d)/98.69 |
| glutamate | βCH^y^H^x^: 2.05(m), **2.12**(m)/29.83, γCH_2_: 2.35(m)/36.26, αCH: 3.75(dd)/57.61 |
| glycine | αCH_2_: **3.55**(s)/44.39 |
| isoleucine | δCH_3_: 0.93(t)/13.89, γ2CH_3_: 1.00(d)/17.43, γ1CH_2_: 1.45(m)/27.20, βCH: 1.98(m)^c^, αCH: 3.67(d)/62.45 |
| lactate | βCH_3_: 1.32(d)/22.90, αCH: 4.11(q)/71.32 |
| leucine | δ^y^CH_3_: 0.95(d)/23.66, δ^x^CH_3_: 0.96(d)/24.68, βCH_2_: 1.71(m)/42.68, γCH: 1.71(m)/26.81, αCH: 3.73(m)/56.36 |
| lysine | γCH_2_: 1.46(m)/24.58, δCH_2_: **1.70**(m)/29.14, βCH_2_: 1.89(m)/32.67, εCH_2_: 3.01(m)/42.09, αCH: 3.75(m)/57.37 |
| methanol | CH_3_: **3.35**(s)/51.72 |
| Mg-EDTA^2-^ | αCH_2_: 2.69(s)/58.06, γCH_2_: 3.22(d)/63.69 |
| myo-inositol | 2-CH: 3.28(t)/77.15, 5-CH: **4.06**(t)/74.95 |
| N-acetyl glycoproteins | NHCOCH_3_: **2.04**(b)/24.88 |
| O-acetyl glycoproteins | OCOCH_3_: 2.07(b)/25.20 |
| phosphatidylcholine | N(CH_3_)_3_: 3.21(b)/56.70 |
| trimethylamine N-oxide | N(CH_3_)_3_: **3.26**(s)/62.30 |
| trimethylamine | N(CH_3_)_3_: **2.89**(s)^c^ |
| tyrosine | εCH: 6.89(m)/118.61, δCH: 7.19(m)/133.45 |
| valine | γ^y^CH_3_: 0.98(d)/19.38, γ^x^CH_3_: 1.03(d)/20.62, βCH: 2.26(m)/31.87, αCH: 3.61(d)/63.19 |
| U1 | **1.12**(d)/21.59, 4.25(m)/56.85 |
| U2 | **2.75**(s)/42.44 |
| U3 | **3.03**(t)/61.04, 3.86(t)/59.23 |
| U4 | **3.78**(s)/53.48 |
| U5 | **2.93**(m)/54.55, 3.14(m)/50.01 |

^a^ The assignments of most metabolites are equivalent to Metabolomics Standard Initiative level 2 (putatively annotated compounds, *i.e.* assignments based upon physicochemical properties and/or spectral similarity with public/commercial spectral libraries but no internal standard used), except for unidentified metabolites U1-U5, which are level 4 (unidentified metabolites).

^b^The resonance assignment of metabolites was based on literature as well as 1D and 2D NMR experiments. ^1^H and ^13^C chemical shifts of metabolites obtained from ^1^H-^13^C heteronuclear experiments are presented. Buckets containing the bolded signals were used for quantification in Tables 2 and 3. Key: s, singlet; d, doublet; dd, double doublet; t, triplet; q, quartet; m, multiplet; b, broad.

^c^ Corresponding ^13^C chemical shifts were not detected by ^1^H-^13^C heteronuclear experiments.
