# Supplementary material for: Impacts of the Callipyge Mutation on Ovine Plasma Metabolites and Muscle Fibre Type
Source: PLoS One. 2014 Jun 17;9(6):e99726. doi: 10.1371/journal.pone.0099726 (PMC4061035; doi:10.1371/journal.pone.0099726)
Supplement: Table S2 — The properties of multivariate statistical analysis models of plasma samples. (DOCX) [file pone.0099726.s006.docx]

**Table S2.** The properties of multivariate statistical analysis models of plasma samples.

| Experiment | Effect | Method | *A* ^a^ | *N* ^b^ | *R^2^X* ^c^ | *R^2^Y* | *Q^2^* | *P* |
| --- | --- | --- | --- | --- | --- | --- | --- | --- |
| CPMG | Outliers | PCA | 10 | 76 | 0.88 |  | 0.72 |  |
|  | Age | PCA | 12 | 73 | 0.86 |  | 0.65 |  |
|  |  | PLS-DA | 3 | 73 | 0.40 | 0.76 | 0.52 |  |
|  |  | OPLS-DA | 1+3+0 | 73 | 0.51 | 0.81 | 0.60 | 2.33·10^-10^ |
|  | Gender | PLS-DA | 2 | 73 | 0.30 | 0.25 | -0.12* |  |
|  |  | OPLS-DA | 1+1+0 | 73 | 0.30 | 0.25 | -0.15* | 1 |
|  | genotype at 8 wks | PCA | 7 | 36 | 0.81 |  | 0.54 |  |
|  |  | PLS-DA | 2 | 36 | 0.30 | 0.73 | -0.18* |  |
|  |  | OPLS-DA | 1+1+0 | 36 | 0.30 | 0.73 | -0.19* | 1 |
|  | genotype at 12 wks | PCA | 8 | 37 | 0.83 |  | 0.47 |  |
|  |  | PLS-DA | 2 | 37 | 0.29 | 0.68 | 0.30 |  |
|  |  | OPLS-DA | 1+1+0 | 37 | 0.29 | 0.68 | 0.33 | 0.01 |
| NOESY | Outliers | PCA | 14 | 76 | 0.96 |  | 0.80 |  |
|  | Age | PCA | 14 | 73 | 0.95 |  | 0.83 |  |
|  |  | PLS-DA | 2 | 73 | 0.33 | 0.58 | 0.32 |  |
|  |  | OPLS-DA | 1+3+0 | 73 | 0.67 | 0.71 | 0.52 | 5.19·10^-8^ |
|  | Gender | PLS-DA | 2 | 73 | 0.37 | 0.15 | -0.15* |  |
|  |  | OPLS-DA | 1+1+0 | 73 | 0.37 | 0.15 | -0.07* | 1 |
|  | genotype at 8 wks | PCA | 8 | 36 | 0.91 |  | 0.68 |  |
|  |  | PLS-DA | 2 | 36 | 0.47 | 0.42 | -0.03* |  |
|  |  | OPLS-DA | 1+1+0 | 36 | 0.47 | 0.42 | -0.02* | 1 |
|  | genotype at 12 wks | PCA | 10 | 37 | 0.94 |  | 0.72 |  |
|  |  | PLS-DA | 3 | 37 | 0.59 | 0.70 | 0.42 |  |
|  |  | OPLS-DA | 1+1+0 | 37 | 0.48 | 0.56 | 0.35 | 0.006 |

^a^ Number of latent components. The notation for OPLS-DA models is: number of joint components + number of *Y*-orthogonal (unique to *X*) components + number of *X*-orthogonal (unique to *Y*) components.

^b^ Number of samples

^c^ *R^2^X* and *R^2^Y* are the fraction of the sum of squares for the selected component representing the variance of *X* and *Y* variables, and *Q^2^* is the predictive ability parameter of the model, which is estimated by cross-validation. OPLS-DA models are further characterized by their *P* value obtained from CV-ANOVA. The *R^2^X*, *R^2^Y*, *Q^2^* and *P* values demonstrate that developmental stage and the Callipyge genotype at 12 weeks of age affected lamb plasma metabolites. The eight negative *Q^2^* values (*) in the table denote invalid PLS-DA and OPLS‑DA models. Thus, genotype at 8 weeks of age and gender have no impact on lamb plasma metabolites.
