# Supplementary material for: Impacts of the Callipyge Mutation on Ovine Plasma Metabolites and Muscle Fibre Type
Source: PLoS One. 2014 Jun 17;9(6):e99726. doi: 10.1371/journal.pone.0099726 (PMC4061035; doi:10.1371/journal.pone.0099726)
Supplement: Table S3 — Microarray analysis of perirenal adipose tissue as a function of Callipyge genotype. (DOCX) [file pone.0099726.s007.docx]

**Table S3.** Microarray analysis of perirenal adipose tissue as a function of Callipyge genotype.

| **Feature ID** | ***Gene symbol*** | **log_2_ (NC/NN)** | ***P*-value ^a^** | **NC - Log_2_ Normalized means** | **NN - Log _2_ Normalized means** |
| --- | --- | --- | --- | --- | --- |
| Bt.17260.2.A1_at | *DCLRE1C* | 3.0465 | 0.0206 | 2.1879 | -0.8587 |
| Bt.19547.1.A1_at | *SYT10* | 2.9026 | 0.0214 | 3.1045 | 0.2020 |
| Bt.26899.1.A1_at | *SLITRK4* | 2.8747 | 0.0100 | 2.5064 | -0.3683 |
| Bt.22985.1.S1_at | *HNF4G* | 2.8550 | 0.0166 | 3.0305 | 0.1755 |
| Bt.26359.1.A1_at | *SLC13A4* | 2.7790 | 0.0291 | 4.5539 | 1.7749 |
| Bt.17722.1.A1_at | *RBL2* | 2.6630 | 0.0065 | 3.5980 | 0.9350 |
| Bt.22305.1.A1_at | *ITPR1* | 2.6115 | 0.0097 | 3.4770 | 0.8655 |
| Bt.17637.3.A1_at | *GADD45A* | 2.6101 | 0.0002 | 4.0342 | 1.4241 |
| Bt.9566.1.S1_at | *CDHR1* | 2.5821 | 0.0011 | 4.6405 | 2.0585 |
| Bt.102.1.S1_at | *MYF5* | 2.5520 | 0.0042 | 3.4264 | 0.8744 |
| Bt.18915.1.A1_at | *LOC284058* | 2.5425 | 0.0463 | 4.1151 | 1.5726 |
| Bt.8363.1.A1_at | *KCNK1* | 2.4836 | 0.0129 | 4.8902 | 2.4066 |
| Bt.17472.1.A1_at | *PB1* | 2.4804 | 0.0280 | 4.6999 | 2.2195 |
| Bt.22194.3.S1_at |  | 2.4625 | 0.0044 | 3.6903 | 1.2278 |
| Bt.19034.1.A1_at | *PHTF1* | 2.4088 | 0.0194 | 4.3061 | 1.8973 |
| Bt.24240.1.S1_at | *DHTKD1* | 2.3773 | 0.0021 | 4.1724 | 1.7951 |
| Bt.25376.1.A1_at | *CSMD3* | 2.3238 | 0.0425 | 4.3173 | 1.9935 |
| Bt.26310.1.A1_at | *SMARCA5* | 2.2833 | 0.0060 | 5.1141 | 2.8309 |
| Bt.18289.1.A1_at | *JUND* | 2.2601 | 0.0252 | 4.5649 | 2.3048 |
| Bt.18603.1.A1_at | *KIAA1012* | 2.2258 | 0.0232 | 3.7166 | 1.4908 |
| Bt.18803.2.A1_at | *XIST* | 2.2245 | 0.0411 | 4.2581 | 2.0336 |
| Bt.5772.1.S1_at | *ERAF* | 2.2216 | 0.0255 | 5.9937 | 3.7721 |
| Bt.13796.1.S1_at | *DKFZP434F122* | 2.2195 | 0.0056 | 4.9016 | 2.6822 |
| Bt.19593.1.A1_at |  | 2.2048 | 0.0395 | 3.7043 | 1.4995 |
| Bt.605.1.S1_at | *MSTN* | 2.2023 | 0.0264 | 5.2104 | 3.0081 |
| Bt.16283.1.A1_at | *LOC92345* | 2.1822 | 0.0024 | 5.5602 | 3.3780 |
| Bt.10996.1.S1_at | *GDPD2* | 2.1640 | 0.0019 | 5.7341 | 3.5700 |
| Bt.10786.1.A1_at | *BMP8B* | 2.1589 | 0.0384 | 4.6316 | 2.4727 |
| Bt.25693.1.S1_at | *PLEKHG2* | 2.1345 | 0.0043 | 3.3615 | 1.2269 |
| Bt.1461.2.A1_at | *RNF7* | 2.1302 | 0.0142 | 2.3456 | 0.2154 |
| Bt.29348.1.A1_at |  | 2.0877 | 0.0280 | 6.2036 | 4.1159 |
| Bt.12300.2.S1_at | *MYH1* | 2.0874 | 0.0500 | 4.0743 | 1.9870 |
| Bt.29682.1.A1_at | *LOC407199* | 2.0807 | 0.0012 | 3.8649 | 1.7841 |
| Bt.13736.3.S1_at | *FLJ22028* | 2.0736 | 0.0458 | 6.0329 | 3.9592 |
| Bt.24965.1.A1_at | *FLJ30092* | 2.0630 | 0.0137 | 4.8085 | 2.7455 |
| Bt.13835.1.S1_at | *MCART2* | 2.0530 | 0.0378 | 3.8529 | 1.7999 |
| Bt.17053.1.A1_at | *KIAA1679* | 2.0026 | 0.0496 | 4.0018 | 1.9991 |
| Bt.3253.1.A1_at | *LOC617871* | 1.9953 | 0.0548 | 4.9057 | 2.9105 |
| Bt.18344.1.A1_at | *STAU1* | 1.9868 | 0.0351 | 1.9234 | -0.0635 |
| Bt.18622.1.A1_at | *RANBP3* | 1.9751 | 0.0244 | 4.0422 | 2.0671 |
| Bt.24851.1.S1_at | *PEX14* | 1.9502 | 0.0366 | 5.6111 | 3.6609 |
| Bt.24990.2.S1_at | *USP21* | 1.9471 | 0.0028 | 4.7572 | 2.8102 |
| Bt.29405.1.A1_at |  | 1.9449 | 0.0301 | 3.9209 | 1.9760 |
| Bt.16408.1.A1_at | *MLLT3* | 1.9295 | 0.0483 | 2.3090 | 0.3795 |
| Bt.23538.2.S1_at | *FADD* | 1.9269 | 0.0343 | 5.1754 | 3.2485 |
| Bt.17170.1.A1_at | *IQCF2* | 1.9167 | 0.0168 | 3.8503 | 1.9336 |
| Bt.29793.1.S1_at |  | 1.9066 | 0.0215 | 3.6847 | 1.7781 |
| Bt.19048.1.A1_at |  | 1.8959 | 0.0004 | 5.5687 | 3.6728 |
| Bt.257.1.S1_at |  | 1.8832 | 0.0012 | 4.9027 | 3.0195 |
| Bt.29568.1.S1_at | *EAF2* | 1.8793 | 0.0466 | 3.6514 | 1.7721 |
| Bt.14040.2.A1_at | *PTGR2* | 1.8730 | 0.0043 | 5.1218 | 3.2487 |
| Bt.24354.1.S1_at | *CSTB* | 1.8718 | 0.0174 | 5.0076 | 3.1357 |
| Bt.18158.1.A1_at | *KIAA2018* | 1.8701 | 0.0279 | 3.0417 | 1.1715 |
| Bt.12806.1.S1_at | *HR44* | 1.8696 | 0.0500 | 4.4095 | 2.5399 |
| Bt.29389.1.S1_at | *CHSY1* | 1.8647 | 0.0150 | 5.0200 | 3.1552 |
| Bt.17795.1.A1_at | *ADAM12* | 1.8586 | 0.0108 | 3.1274 | 1.2688 |
| Bt.20925.1.S1_at | *BOLA-DQB* | 1.8470 | 0.0042 | 4.6684 | 2.8213 |
| Bt.19081.1.A1_at | *LAMC1* | 1.8316 | 0.0486 | 4.0628 | 2.2312 |
| Bt.23874.1.A1_at |  | 1.8295 | 0.0002 | 4.1490 | 2.3195 |
| Bt.16650.1.A1_at |  | 1.8254 | 0.0393 | 5.1496 | 3.3242 |
| Bt.19712.1.A1_at | *CENPC1* | 1.8221 | 0.0452 | 3.5123 | 1.6901 |
| Bt.23936.1.A1_at | *MPP6* | 1.8104 | 0.0391 | 4.5157 | 2.7053 |
| Bt.28386.1.A1_at | *BRWD1* | 1.8013 | 0.0103 | 4.1999 | 2.3987 |
| Bt.29686.1.A1_at | *FGFR2* | 1.8002 | 0.0223 | 4.4096 | 2.6094 |
| Bt.28042.1.S1_at | *LNP1* | 1.7940 | 0.0510 | 4.7594 | 2.9654 |
| Bt.3686.1.S1_at | *IL6* | 1.7903 | 0.0454 | 3.5564 | 1.7660 |
| Bt.17473.2.S1_at | *RPE* | 1.7780 | 0.0353 | 4.7944 | 3.0164 |
| Bt.2464.1.A1_at | *PPP1R12C* | 1.7651 | 0.0400 | 5.2784 | 3.5133 |
| Bt.18475.2.A1_at | *STX3A* | 1.7638 | 0.0514 | 2.5638 | 0.8000 |
| Bt.13996.2.S1_at |  | 1.7546 | 0.0298 | 4.0094 | 2.2548 |
| Bt.19197.1.A1_at | *G6PC* | 1.7466 | 0.0237 | 4.5687 | 2.8221 |
| Bt.15797.1.S1_at | *HSPC152* | 1.7455 | 0.0053 | 4.4669 | 2.7214 |
| Bt.28511.2.S1_at | *ART1* | 1.7311 | 0.0031 | 4.3652 | 2.6341 |
| Bt.18295.1.A1_at | *PTK2* | 1.7154 | 0.0416 | 2.8261 | 1.1107 |
| Bt.29783.1.A1_at |  | 1.7154 | 0.0445 | 3.1140 | 1.3986 |
| Bt.16271.2.A1_at | *CLEC4E* | 1.6790 | 0.0312 | 2.6285 | 0.9494 |
| Bt.18116.1.S1_at | *PARP12* | 1.6684 | 0.0543 | 4.3284 | 2.6600 |
| Bt.5914.1.S1_at | *PRRG3* | 1.6154 | 0.0152 | 4.1831 | 2.5677 |
| Bt.27714.1.A1_at |  | 1.6148 | 0.0543 | 2.8393 | 1.2245 |
| Bt.25985.1.A1_at | *PROK2* | 1.6133 | 0.0290 | 4.3837 | 2.7705 |
| Bt.2357.1.S1_at | *LGI1* | 1.6034 | 0.0441 | 3.6078 | 2.0045 |
| Bt.6939.1.S1_at |  | 1.6012 | 0.0211 | 4.7738 | 3.1726 |
| Bt.27504.1.A1_at |  | 1.5987 | 0.0110 | 4.5312 | 2.9326 |
| Bt.29863.1.A1_s_at | *SLC35D1* | 1.5714 | 0.0161 | 4.3808 | 2.8093 |
| Bt.10538.1.A1_at | *RNF165* | 1.5666 | 0.0216 | 1.2878 | -0.2788 |
| Bt.29765.1.S1_at | *TRPV6* | 1.5113 | 0.0389 | 1.9117 | 0.4004 |
| Bt.28415.1.S1_at | *PAMCI* | 1.5109 | 0.0291 | 3.6285 | 2.1176 |
| Bt.26405.1.A1_at |  | 1.5049 | 0.0402 | 3.9703 | 2.4654 |
| Bt.18857.1.A1_at |  | 1.4649 | 0.0191 | 1.6887 | 0.2238 |
| Bt.29173.1.A1_at |  | 1.4538 | 0.0169 | 2.4127 | 0.9589 |
| Bt.305.1.S1_at | *PKP1* | 1.4116 | 0.0330 | 3.9953 | 2.5838 |
| Bt.22950.1.A1_at | *CRISP-1* | 1.3980 | 0.0158 | 2.0294 | 0.6314 |
| Bt.29842.1.A1_at | *SLC19A1* | 1.3792 | 0.0199 | 4.1019 | 2.7228 |
| Bt.19612.1.S1_at | *PGM3* | 1.3565 | 0.0072 | 3.7185 | 2.3620 |
| Bt.568.1.S1_at | *IBSP* | 1.3516 | 0.0176 | 3.6998 | 2.3482 |
| Bt.16169.1.A1_at | *LMO3* | 1.3255 | 0.0406 | 3.8612 | 2.5358 |
| Bt.18649.2.A1_at | *TMEM216* | 1.2838 | 0.0161 | 3.5664 | 2.2826 |
| Bt.16984.1.S1_at | *LOC441459* | 1.2773 | 0.0271 | 3.6879 | 2.4106 |
| Bt.23885.1.A1_at |  | 1.2648 | 0.0412 | 1.8995 | 0.6347 |
| Bt.311.1.S1_at | *ELA2B* | 1.2415 | 0.0541 | 2.9482 | 1.7067 |
| Bt.24180.1.A1_at |  | 1.2055 | 0.0226 | 2.8025 | 1.5970 |
| Bt.28572.1.S1_at | *GFI1* | 1.1880 | 0.0111 | 3.1757 | 1.9877 |
| Bt.9565.1.S1_at | *LOC404073* | 1.0576 | 0.0294 | 2.3218 | 1.2642 |
| Bt.15801.1.A1_at | *LOC506943* | -0.6736 | 0.0347 | 1.2950 | 1.9686 |
| Bt.18359.1.A1_at |  | -0.9239 | 0.0472 | 1.7345 | 2.6584 |
| Bt.11474.1.A1_at | *MLL5* | -0.9688 | 0.0220 | 1.2220 | 2.1907 |
| Bt.5038.3.S1_a_at | *FGF1* | -1.0761 | 0.0349 | 1.2006 | 2.2767 |
| Bt.5848.2.S1_at | *ATP2A1* | -1.2671 | 0.0490 | 1.0560 | 2.3231 |
| Bt.9145.1.A1_at | *DXS9879E* | -1.2851 | 0.0039 | 2.4260 | 3.7111 |
| Bt.29134.1.S1_at | *IFRD2* | -1.2868 | 0.0206 | 2.5197 | 3.8065 |
| Bt.24780.1.S1_at | *ARID5B* | -1.2923 | 0.0297 | 2.1625 | 3.4547 |
| Bt.21193.1.S1_at | *CBLC* | -1.3315 | 0.0117 | 2.4950 | 3.8265 |
| Bt.7619.1.S1_at | *TTYH1* | -1.3507 | 0.0043 | 2.6142 | 3.9650 |
| Bt.17622.1.A1_at | *FLJ13868* | -1.3997 | 0.0492 | 2.7731 | 4.1728 |
| Bt.16027.1.S1_at | *LEPR* | -1.4029 | 0.0488 | 2.0754 | 3.4782 |
| Bt.8274.2.S1_at | *HIPK4* | -1.4413 | 0.0088 | 2.5185 | 3.9597 |
| Bt.21654.1.S1_at |  | -1.4603 | 0.0140 | 2.4080 | 3.8683 |
| Bt.18120.1.A1_at | *DDX5* | -1.4785 | 0.0129 | 0.6884 | 2.1669 |
| Bt.25243.1.A1_at |  | -1.4827 | 0.0205 | 2.3187 | 3.8014 |
| Bt.28998.1.A1_at |  | -1.5078 | 0.0415 | 3.0090 | 4.5168 |
| Bt.23800.1.A1_at | *PPP4R1L* | -1.5374 | 0.0439 | 0.8883 | 2.4256 |
| Bt.13645.2.S1_at |  | -1.5592 | 0.0263 | 2.2592 | 3.8184 |
| Bt.22162.1.S1_at | *CXCR4* | -1.5663 | 0.0504 | 1.4265 | 2.9928 |
| Bt.26477.1.A1_at | *CCL3* | -1.5746 | 0.0279 | 1.5200 | 3.0947 |
| Bt.6435.1.A1_at | *PIP5K1C* | -1.5758 | 0.0037 | 3.0888 | 4.6646 |
| Bt.25491.1.A1_at | *DNAH5* | -1.5949 | 0.0164 | 3.0313 | 4.6263 |
| Bt.17670.1.A1_at | *GPRIN3* | -1.6077 | 0.0345 | 2.3986 | 4.0063 |
| Bt.25967.1.A1_at |  | -1.6079 | 0.0306 | 1.9082 | 3.5161 |
| Bt.19356.1.A1_at | *BHMT* | -1.6513 | 0.0414 | 3.0431 | 4.6944 |
| Bt.17956.1.A1_at | *STAT1* | -1.6807 | 0.0497 | 2.7245 | 4.4052 |
| Bt.483.1.S2_at | *CLDN16* | -1.6834 | 0.0132 | 1.2006 | 2.8840 |
| Bt.21887.1.A1_at | *PBX1* | -1.6857 | 0.0218 | 3.1884 | 4.8742 |
| Bt.18208.1.A1_at | *ZBTB32* | -1.6880 | 0.0186 | 3.1607 | 4.8486 |
| Bt.4404.1.A1_at | *TRY6* | -1.6955 | 0.0381 | 2.6483 | 4.3438 |
| Bt.29597.1.A1_at |  | -1.7085 | 0.0292 | 2.2574 | 3.9658 |
| Bt.27850.1.S1_at | *LOC535353* | -1.7328 | 0.0139 | 3.2210 | 4.9538 |
| Bt.28255.1.S1_at | *LOC783222* | -1.7376 | 0.0198 | 1.7517 | 3.4893 |
| Bt.11736.1.A1_at |  | -1.7447 | 0.0094 | 2.3917 | 4.1364 |
| Bt.28536.1.S1_at | *LOC783151* | -1.7479 | 0.0141 | 3.3680 | 5.1160 |
| Bt.191.1.S2_at | *IL1A* | -1.7612 | 0.0330 | 3.1322 | 4.8934 |
| Bt.21117.1.S1_at |  | -1.7628 | 0.0532 | 2.0489 | 3.8116 |
| Bt.27606.1.A1_at |  | -1.7848 | 0.0079 | 3.2362 | 5.0210 |
| Bt.9098.1.A1_at | *DDX58* | -1.7921 | 0.0361 | 2.5681 | 4.3602 |
| Bt.2167.1.S1_at | *RAB27B* | -1.8106 | 0.0254 | 2.2153 | 4.0258 |
| Bt.25443.1.A1_at | *SLC6A1* | -1.8249 | 0.0453 | -0.4115 | 1.4133 |
| Bt.17636.1.A1_s_at | *C5orf18* | -1.8469 | 0.0480 | 1.8042 | 3.6511 |
| Bt.26937.1.S1_at | *LY86* | -1.8523 | 0.0519 | 3.4641 | 5.3164 |
| Bt.27598.1.A1_at | *PAK7* | -1.8833 | 0.0235 | 1.9494 | 3.8327 |
| Bt.360.1.S1_at | *MBL2* | -1.8939 | 0.0380 | 0.9078 | 2.8018 |
| Bt.17660.1.A1_at |  | -1.8992 | 0.0152 | 1.6492 | 3.5484 |
| Bt.16805.1.A1_at | *C18orf17* | -1.9301 | 0.0518 | 0.9358 | 2.8659 |
| Bt.26224.1.S1_at | *BAD* | -1.9338 | 0.0010 | 3.8080 | 5.7418 |
| Bt.13003.15.S1_at |  | -1.9348 | 0.0035 | 2.0738 | 4.0086 |
| Bt.26616.1.S1_at | *LOC519307* | -1.9387 | 0.0311 | 2.0146 | 3.9532 |
| Bt.18576.1.S1_at | *RUTBC1* | -1.9434 | 0.0020 | 3.2567 | 5.2001 |
| Bt.27574.1.A1_at |  | -1.9586 | 0.0256 | 3.1340 | 5.0926 |
| Bt.29779.1.S1_s_at | *CYCS* | -1.9958 | 0.0214 | 1.6260 | 3.6218 |
| Bt.22022.1.A1_at | *LOC511424* | -2.0008 | 0.0310 | 2.4420 | 4.4428 |
| Bt.1343.1.S1_at | *SLC16A9* | -2.0247 | 0.0299 | 3.1116 | 5.1363 |
| Bt.15388.1.A1_at |  | -2.0485 | 0.0311 | 1.2736 | 3.3220 |
| Bt.18545.1.A1_at | *RAB22A* | -2.0696 | 0.0441 | 3.0533 | 5.1229 |
| Bt.29614.1.A1_at | *LAMC2* | -2.0796 | 0.0195 | 1.0882 | 3.1679 |
| Bt.29718.1.A1_a_at | *SEPP1* | -2.1098 | 0.0274 | 1.4585 | 3.5684 |
| Bt.7003.1.S1_at | *LPXN* | -2.1389 | 0.0202 | 2.9270 | 5.0659 |
| Bt.9106.1.A1_at | *GAN* | -2.1475 | 0.0015 | 2.6102 | 4.7578 |
| Bt.11630.1.A1_at |  | -2.1492 | 0.0270 | 2.5394 | 4.6886 |
| Bt.28004.2.A1_x_at | *FLJ22405* | -2.1717 | 0.0189 | 2.0811 | 4.2528 |
| Bt.2810.1.A1_at | *CDKN3* | -2.2197 | 0.0020 | 2.2659 | 4.4856 |
| Bt.17635.1.A1_at | *ELOVL4* | -2.2203 | 0.0528 | 2.9761 | 5.1964 |
| Bt.29681.1.S1_at | *PRP6* | -2.2676 | 0.0104 | 0.7762 | 3.0437 |
| Bt.18902.1.A1_at | *ATRN* | -2.2683 | 0.0280 | 1.2996 | 3.5679 |
| Bt.17679.1.A1_at | *LOC441155* | -2.2774 | 0.0029 | 1.3059 | 3.5833 |
| Bt.27616.1.A1_at | *ZIC3* | -2.2839 | 0.0524 | 0.9365 | 3.2204 |
| Bt.20837.2.A1_at | *PPFIA1* | -2.2972 | 0.0471 | 1.3676 | 3.6648 |
| Bt.1796.1.S1_at | *MPG* | -2.3095 | 0.0047 | 3.0219 | 5.3314 |
| Bt.18628.1.A1_at | *NDUFAF1* | -2.3104 | 0.0130 | 0.1207 | 2.4311 |
| Bt.11587.3.A1_a_at | *SPAG5* | -2.3128 | 0.0111 | 3.4945 | 5.8074 |
| Bt.27554.1.A1_at |  | -2.3439 | 0.0408 | 3.1452 | 5.4890 |
| Bt.19856.1.A1_at | *LOC132706* | -2.3646 | 0.0184 | 1.4358 | 3.8004 |
| Bt.11589.1.A1_at | *SERPIND1* | -2.3925 | 0.0238 | 1.3357 | 3.7282 |
| Bt.29462.1.S1_at | *CDCA2* | -2.4015 | 0.0159 | 2.3481 | 4.7496 |
| Bt.18184.1.A1_at | *RABEP1* | -2.4115 | 0.0115 | 0.2731 | 2.6846 |
| Bt.18539.1.A1_at |  | -2.4539 | 0.0047 | 0.9936 | 3.4475 |
| Bt.14159.1.A1_at | *RPL7L1* | -2.4636 | 0.0161 | 2.0297 | 4.4933 |
| Bt.25375.1.A1_at |  | -2.5286 | 0.0145 | 2.3880 | 4.9166 |
| Bt.27560.1.A1_at | *DKFZp434A128* | -2.5409 | 0.0025 | 1.3617 | 3.9026 |
| Bt.19326.1.A1_at |  | -2.5559 | 0.0166 | 1.1209 | 3.6767 |
| Bt.6172.1.A1_at | *PCDH19* | -2.5891 | 0.0200 | 3.6934 | 6.2824 |
| Bt.16692.1.A1_at | *ETFA* | -2.6088 | 0.0394 | 0.2281 | 2.8369 |
| Bt.24592.1.S1_at | *NRN1* | -2.6285 | 0.0020 | 1.3316 | 3.9601 |
| Bt.26578.1.S1_at | *SLC13A3* | -2.6429 | 0.0011 | 1.9111 | 4.5540 |
| Bt.25024.1.S1_at | *CAMK2N2* | -2.6675 | 0.0051 | 3.0155 | 5.6829 |
| Bt.23829.1.A1_at | *KIAA1155* | -2.8008 | 0.0510 | 1.7976 | 4.5984 |
| Bt.18640.1.A1_at | *VTI1A* | -2.8069 | 0.0051 | 1.9078 | 4.7148 |
| Bt.18542.1.A1_at | *TCF4* | -2.8129 | 0.0190 | 0.9610 | 3.7739 |
| Bt.20722.1.A1_at | *GLI3* | -2.8345 | 0.0095 | 0.7378 | 3.5723 |
| Bt.25291.1.A1_at | *UNQ6077* | -2.9276 | 0.0270 | -0.1033 | 2.8244 |
| Bt.17516.1.A1_at | *KIAA1126* | -3.0604 | 0.0128 | 0.7145 | 3.7749 |
| Bt.12283.1.A1_at | *DIRAS3* | -3.0645 | 0.0489 | 3.8071 | 6.8716 |
| Bt.160.2.S1_x_at | *DSC1* | -3.2596 | 0.0068 | -0.8023 | 2.4573 |
| Bt.5130.2.A1_x_at | *ALB* | -3.6805 | 0.0317 | 2.0811 | 5.7616 |
| Bt.5130.1.A1_a_at | *ALB* | -3.8264 | 0.0319 | 2.3512 | 6.1776 |
| Bt.17047.1.A1_at | *ELAVL4* | -4.3990 | 0.0388 | 0.8448 | 5.2438 |

^a^ *P*-values are uncorrected for multiple testing. No genes were significant after multiple testing corrections.
